# Deubiquitylating Enzymes in Cancer and Immunity

**DOI:** 10.1002/advs.202303807

**Published:** 2023-10-27

**Authors:** Jiang Ren, Peng Yu, Sijia Liu, Ran Li, Xin Niu, Yan Chen, Zhenyu Zhang, Fangfang Zhou, Long Zhang

**Affiliations:** ^1^ The Eighth Affiliated Hospital Sun Yat‐sen University Shenzhen 518033 P. R. China; ^2^ Zhongshan Institute for Drug Discovery Shanghai Institute of Materia Medica Chinese Academy of Sciences Zhongshan Guangdong P. R. China; ^3^ International Biomed‐X Research Center Second Affiliated Hospital of Zhejiang University School of Medicine Zhejiang University Hangzhou P. R. China; ^4^ Key Laboratory of Precision Diagnosis and Treatment for Hepatobiliary and Pancreatic Tumor of Zhejiang Province Hangzhou 310058 China; ^5^ MOE Laboratory of Biosystems Homeostasis & Protection and Innovation Center for Cell Signaling Network Life Sciences Institute Zhejiang University Hangzhou 310058 P. R. China; ^6^ Department of Neurosurgery The First Affiliated Hospital of Zhengzhou University Zhengzhou Henan 450003 P. R. China; ^7^ Institutes of Biology and Medical Science Soochow University Suzhou 215123 P. R. China; ^8^ Cancer Center Zhejiang University Hangzhou Zhejiang 310058 P. R. China

**Keywords:** cancer, deubiquitylating enzymes (DUBs), immunity, therapeutic approach

## Abstract

Deubiquitylating enzymes (DUBs) maintain relative homeostasis of the cellular ubiquitome by removing the post‐translational modification ubiquitin moiety from substrates. Numerous DUBs have been demonstrated specificity for cleaving a certain type of ubiquitin linkage or positions within ubiquitin chains. Moreover, several DUBs perform functions through specific protein–protein interactions in a catalytically independent manner, which further expands the versatility and complexity of DUBs’ functions. Dysregulation of DUBs disrupts the dynamic equilibrium of ubiquitome and causes various diseases, especially cancer and immune disorders. This review summarizes the Janus‐faced roles of DUBs in cancer including proteasomal degradation, DNA repair, apoptosis, and tumor metastasis, as well as in immunity involving innate immune receptor signaling and inflammatory and autoimmune disorders. The prospects and challenges for the clinical development of DUB inhibitors are further discussed. The review provides a comprehensive understanding of the multi‐faced roles of DUBs in cancer and immunity.

## Graphical abstract

This review presents the fundamental understanding of deubiquitinating enzymes (DUBs) and then outlines the mechanisms and multi‐role of DUBs in determining the occurrence and development of tumor and immune disorders. DUBs in the crosstalk between cancer and immune response is then presented. The therapeutic potential and clinical translation of DUB inhibitors are also summarized.

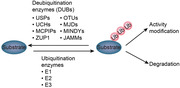

## 1 Introduction

**Figure 1:**
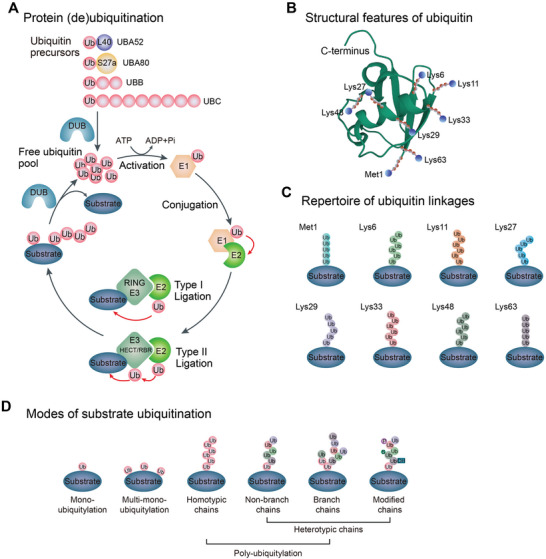
The ubiquitination cascade and the complexity of ubiquitin modifications. A) Schematic showing the key events in ubiquitination and deubiquitination. Ubiquitin (Ub) is produced by cleavage of Ub precursors via deubiquitylating enzymes (DUBs). Ubiquitin precursors include four different genes: UBB and UBC genes code for a polyubiquitin precursor and UBA52 and UBA80 genes code for a single copy of Ub fused to the ribosomal proteins L40 (L) and S27a (S), respectively. Ubiquitin is activated by the E1‐activating enzyme in an ATP‐dependent manner. The E2‐conjugating enzyme catalyzes the conjugation of E1‐bound Ub to itself. Finally, E3 ligase directly (RING E3 ligases) or indirectly (HECT/RBR E3 ligases) transfers E2‐bound Ub to a substrate, forming a thioester linkage between the carboxyl group of glycine in Ub and the ε‐amino group of lysine in the substrate. Ubiquitin molecules are removed from substrates by DUBs and recycled. B) Schematic representation of the structure of Ub. Stick representation showing the seven Lys residues (Lys6, Lys11, Lys27, Lys29, Lys33, Lys48, and Lys63) and Met1 of Ub (Protein Data Bank ID: 1UBQ^[^ ^612^ ^]^), and amino groups that are modified with Ub during the formation of the chain are marked with blue spheres. C) The repertoire of ubiquitin linkages, Met1, and seven Lys residues in Ub can form specific chain linkages with distinct conformations and exert specific (or unknown) functions. D) A substrate can be modified by mono‐, multi‐mono‐ or poly‐ubiquitin. Ubiquitin chains are colored according to linkage type, and each type of chain represents a distinct posttranslational modification. Polyubiquitin includes homotypic chains and heterotypic chains. Heterotypic chains are either branched or modified, such as SUMOylation (S), phosphorylation (P), or hydroxylation (OH), etc.

Protein ubiquitination is a dynamic and reversible posttranslational modification (PTM), wherein one or more ubiquitin protein (Ub) with 76 amino acids is covalently attached to a substrate protein. This modification occurs on a diverse range of cellular proteins in numerous cellular processes.^[^ ^1^ ^]^ Ubiquitin is coded by four different genes in *Homo sapiens*: ubiquitin A‐52 residue ribosomal protein fusion product 1 (UBA52) and ribosomal protein S27a (UBA80) code for a single ubiquitin fused at the C‐terminus to the ribosomal proteins L40 and S27a, respectively. Ubiquitin B (UBB), and ubiquitin C (UBC) genes are polyubiquitin precursor presented with tandem repeats.^[^ ^2^ ^]^ The ubiquitination process is a cascade reaction involving three types of enzymes: Ub‐activating enzymes (E1s), Ub‐conjugating enzymes (E2s), and Ub ligase enzymes (E3s).^[^ ^3^ ^]^ Ub is activated by E1 in an ATP‐dependent manner by forming a thioester bond between the Cys of its active site and glycine C‐terminal carboxyl group of Ub. Subsequently, Ub is bound to E2 through transthiolation and then covalently conjugated to the amino group of Lys residue of a substrate protein by E3 Ub ligases.^[^ ^4^ ^]^ There are currently four E3 subtypes identified: homologous with E6‐associated protein C‐terminus (HECT)‐, really interesting new gene (RING)‐, U‐box‐, and RING‐ between‐RING (RBR)‐type. RING‐ and U‐box‐type E3s directly facilitate Ub transfer from E2 to the substrate protein. Whereas, HECT‐ and RBR‐type E3s can form a thioester bond between their active site cysteine and Ub before transferring Ub to the substrate protein (**Figure** **1**A).^[^ ^5^, ^6^ ^]^

Ubiquitin function mainly relies on seven Lys residues (Lys6, Lys11, Lys27, Lys29, Lys33, Lys48, and Lys63) and an N‐terminal methionine (Met1) (Figure 1B), which facilitates intricate ubiquitination linkages (Figure 1C).^[^ ^7^ ^]^ Ub is attached as a monomer or multiple mono‐ubiquitin adducts to the same or different residues of substrate proteins. To add complexity, poly‐ubiquitination can generate homotypic chains, or heterotypic, or branched chains. What is more, ubiquitin moieties can also be modified by other PTMs (Figure 1D).^[^ ^8^, ^9^ ^]^ Each linkage reconciles distinctive signaling pathways to shape the fate of substrate proteins.^[^ ^10^, ^11^ ^]^ The most typical Lys48‐ or Lys63‐linked chains perform either direct proteasomal degradation of substrate proteins through the ubiquitin‐proteasome system (UPS) or non‐degradative roles in administering cellular localization, protein‐protein interactions, and signaling transduction.^[^ ^10^, ^12^ ^]^ In contrast, the remaining ‘atypical’ linkages are less studied till now. Generally, Met1‐linked linear chains participate in inflammatory processes and apoptosis. Lys6‐linkages are implicated in DNA damage response. Lys11‐linkages have been reported to regulate proteasomal degradation, cell cycle, or membrane trafficking. Lys27‐linkages play a role in protein secretion, DNA damage repair, and mitochondrial damage response. Lys29‐linkages are associated with proteasomal degradation, innate immune response, and AMP‐activated protein kinase (AMPK) signaling regulation. Lys33‐linkages can influence innate immune response and intracellular trafficking.^[^ ^6^, ^9^ ^]^

Ubiquitinated substrates are acknowledged by a myriad of proteins involving ubiquitin‐binding domains (UBDs), which interrelate with the surface patches on Ub.^[^ ^13^ ^]^ Ubiquitination is reversed by peptidases termed deubiquitylating enzymes or deubiquitinases (DUBs) through hydrolysis of linkages between Ub moieties or between Ub and substrate.^[^ ^14^, ^15^ ^]^ DUBs trim Ub molecules from substrates to produce a free Ub pool which guarantees Ub recycling. Ubiquitination and deubiquitination regulate many aspects of human cell biology and physiology. Dysregulation in these processes leads to various clinical implications, including cancer and infection diseases.^[^ ^16^, ^17^ ^]^ This review presents the fundamental understanding of DUBs, and then outlines the mechanisms and Janus‐faced roles of DUBs in determining the occurrence and development of tumor and immune disorders. We also discuss the therapeutic potential and clinical translation of DUB inhibitors based on the cellular mechanisms.

## 2 The Characteristics of DUBs

### 2.1 DUB Superfamily and Structure

**Figure 2:**
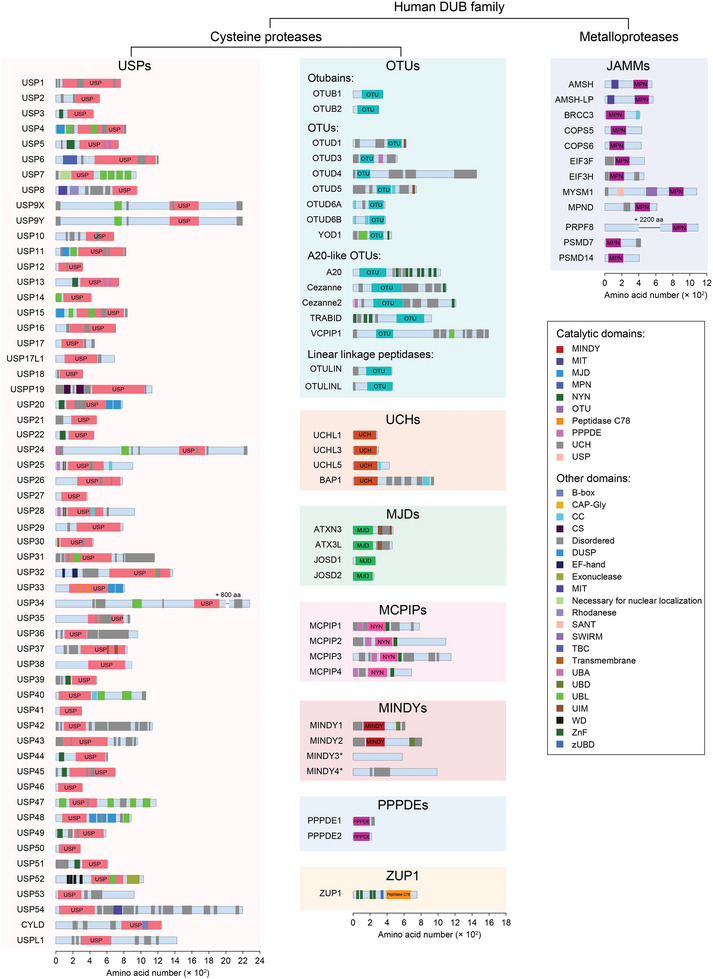
Subfamilies and domain structure of the human DUBs. The human DUBs are classified into six families of cysteine proteases, including ubiquitin‐specific proteases (USPs), ovarian tumor proteases (OTUs), ubiquitin C‐terminal hydrolases (UCHs), Machado–Joseph domain‐containing proteases (MJDs) or Josephins, monocyte chemotactic protein‐inducing proteins (MCPIPs), the motif interacting with ubiquitin (MIU)‐containing novel DUB family (MINDYs), permutated papain fold peptidase of DsRNA viruses and eukaryotes (PPPDEs), zinc finger containing Ubiquitin Peptidase 1 (ZUP1) or zinc finger with UFM1‐specific peptidase domain protein/C6orf113/ZUP1 (ZUFSP); and one metalloprotease subfamily, i.e., JAB1/MPN/MOV34 metalloenzymes (JAMMs, also known as MPN+). CAP‐Gly, cytoskeleton‐associated protein‐glycine‐rich; CC, coiled‐coil; CS, CHORD‐containing proteins and SGT1; MIT, the microtubule interacting and trafficking; MPN, Mpr1/Pad1 N‐terminal; MIT, the microtubule interacting and trafficking; NYN, Nedd4‐BP1, YacP nucleases; SANT, SWI3, ADA2, N‐CoR and TFIIIB DNA‐binding; SWIRM, SWI3P, RSC8P and MOIRA; TBC, Tre2–Bub2–Cdc16; UBA, ubiquitin‐associated; UBD, ubiquitin‐binding domain; UBL, Ubiquitin‐like; UIM, ubiquitin‐interacting motif; ZnF, zinc finger. Numbers with a "+" indicate the number of amino acids is not shown in the sequence. "*" indicates there is no catalytic domain annotated.

More than 100 DUBs are identified in humans and classified into 9 superfamilies according to the sequence and domain conservation: ubiquitin‐specific proteases (USPs), ovarian tumor proteases (OTUs), ubiquitin C‐terminal hydrolases (UCHs), Machado‐Joseph domain‐containing proteases (MJDs, also known as Josephins), JAMM/MPN domain‐associated Zn‐depend metalloproteases (JAMMs, also known as MPN+), the motif interacting with ubiquitin (MIU)‐containing novel DUB family (MINDYs), monocyte chemotactic protein‐induced proteins (MCPIPs), permuted papain fold peptidases of dsRNA viruses and eukaryotes (PPPDEs), and zinc finger (ZnF) containing ubiquitin peptidase 1 (ZUP1) (**Figure** **2**).

DUBs generally contain one or more binding domains for substrate recognition, for example, the ZnF motif, ubiquitin‐like domain (UBL), coiled‐coil (CC) domain, and ubiquitin‐interacting motif (UIM), etc., which cooperates with an indispensable catalytic domain to precisely govern hydrolysis. According to the hydrolysis mechanism of Ub chains, the DUB families identified so far mostly belong to cysteine proteases, except the JAMM family which is considered as zinc‐metalloprotease (Figure 2, Figure 4A).^[^ ^18^, ^19^ ^]^ The DUBs with cysteine protease activity commonly exhibit a catalytic pocket comprising cysteine, histidine, and an acidic residue. The JAMM family DUBs are characterized as Zinc (Zn)‐dependent metalloproteases, coordinating zinc ions with aspartate, histidine, and serine residues to facilitate the recruitment of water molecules and initiate hydrolysis of isopeptide bonds.^[^ ^20^, ^21^, ^22^, ^23^ ^]^

**Figure 3:**
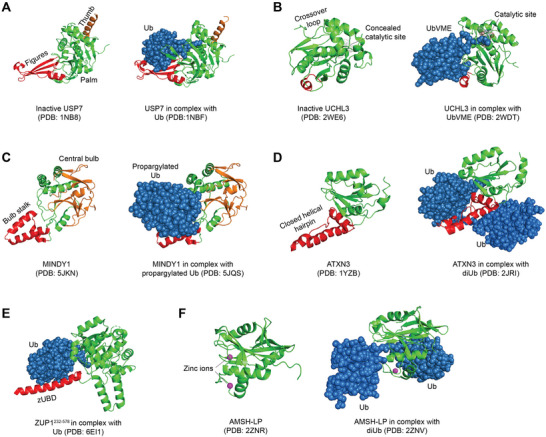
Representative crystal structures of DUBs in cartoon. The crystal structures of USP7 (A), UCHL3 (B), MINDY1 (C), ATXN3 (D), ZUP1^231–576^ (E), AMSH‐LP (F) in the absence (left panel) or presence (right panel) of Ub substrates/inhibitors were adapted from protein data bank (PDB) using PyMOL software.

The first USP structure study was USP7 in the presence and absence of substrate. USP7 exhibits a conserved three‐domain architecture, consisting of fingers, palm, and thumb. The leaving ubiquitin moiety is specifically coordinated by the fingers, positioning its C terminus within the active site situated between the palm and the thumb. The binding of ubiquitin aldehyde induces a significant conformational change in the active site, resulting in the realignment of catalytic triad residues to facilitate catalysis (**Figure** **3**A).^[^ ^24^ ^]^ Based on the analogy with inhibitor complexes of papain‐like enzymes, researchers proposed a model to elucidate the binding mechanism of UCH DUBs to ubiquitinated substrate. The unliganded structure of UCHL3 revealed that its active site cleft is concealed by two distinct segments of the enzyme, implying a regulatory mechanism to restrict non‐specific hydrolysis. Substrate binding leads to a conformational change. Ubiquitin vinyl methyl ester (UbVME, an inhibitor) forms a covalent adduct with the catalytic active site cysteine of UCHL3 (Figure 3B).^[^ ^25^, ^26^ ^]^

The crystal structure study of the N‐terminal OTU deubiquitinase domain of A20 elucidated distinct characteristics compared to other DUBs, but still having a similar essential catalytic core. The analysis of conserved surface regions enables the prediction of ubiquitin‐binding sites for both the proximal and distal ubiquitin molecules. In addition, structural and biochemical analysis revealed a novel architectural arrangement of the catalytic triad that was potentially found in a subset of OTU domains including Cezanne and TRABID.^[^ ^27^ ^]^ The crystal structure of MINDY‐1 in complex with propargylated Ub reveals conformational changes that reposition the active site for catalysis. MINDY1 exhibits a preference for cleaving lengthy polyUb chains and operates by selectively trimming the chains from the distal ends (Figure 3C).^[^ ^28^ ^]^ The significant discovery of MJD DUBs, particularly ATXN3, demonstrated selective Ub esterase activity implying their specialized role in specifically modifying non‐lysine ubiquitinated substrates (Figure 3D).^[^ ^29^ ^]^ ZUP1, the sole human member of the deubiquitinase family, harbors multiple UBDs responsible for its specific action on K63‐linked chains.^[^ ^30^ ^]^ MINDYs and ZUP1 family members were newly identified, but their cellular function is poorly understood (Figure 3E).^[^ ^28^ ^]^ PPPDE1 is also a newly discovered deubiquitinase enzyme that belongs to the cysteine isopeptidase family, and it plays a regulatory role in ubiquitin activity through its binding affinity for both K48 and K63 linkages.^[^ ^31^ ^]^

The JAMM metalloprotease structures represent the sole current instance in which a deubiquitinating enzyme has been crystallized with a ubiquitin chain bound across its active site. The JAMM motif coordinates two zinc ions, with one of them serving as a catalyst to activate a water molecule for attacking the isopeptide bond. The amino group is subsequently liberated from the charged catalytic intermediate through a mechanism akin to that of cytidine deaminase (Figure 3F).^[^ ^32^ ^]^

Generally, DUB's catalytic activity can be evaluated by a method based on the hydrolysis of the substrate conjugated with ubiquitin 7‐amido‐4‐methylcoumarin (Ub‐AMC) and the determination of fluorogenic substrate AMC in vitro. However, this method is subject to the throughput range of identification, and has not been widely applied to identify DUBs on a large scale. Additionally, whether other types of proteases, such as serine or asparate, execute DUB‐like functions might discover novel DUB subclasses. The development of innovative technology is extremely necessary for the identification of DUB superfamily and the exploration of their diverse functions in the future.^[^ ^33^, ^34^ ^]^

### 2.2 The Ubiquitin Linkage Specificity of DUBs

**Figure 4:**
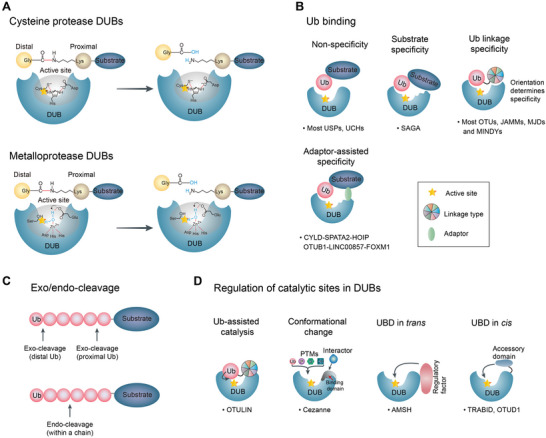
Catalytic DUB mechanisms of cleavage and recognition. A) Cysteine protease DUBs commonly include a catalytic pocket composed of cysteine, histidine, and an acidic residue. JAMM family DUBs are Zinc (Zn)‐dependent metalloproteases. A Zn atom is essential for the active site that is coordinated by conserved catalytic residues and a water molecule. Red represents a fragile isopeptide. B) Principles of substrate recognition and linkage specificity according to a variety of Ub binding and regulation catalytic sites of DUBs. Most members of the USP and UCL subfamilies have nonspecific interactions with the substrate (without a motif). Conversely, SAGA has substrate specificity through motif interactions with the substrate protein. Ub linkage specificity is acquired so that only one linkage type is identified by Ub‐binding sites to catalyze DUB activity. Many members of OTU‐, JAMM‐, Josephin‐, and MINDY families have linkage specificity. In addition, some DUBs recognize targeted proteins with the assistance of adaptors or scaffolds. C) The characteristics of Ub‐binding sites in DUBs determine whether polyubiquitin is cleaved from the distal‐ or the proximal end (exo‐cleavage) or within the en bloc chain (endo‐cleavage). D) Regulation of catalytic sites in DUBs. Ub complements the enzymatic activity by directly participating in the active site, and other post‐translational modifications (SUMOylation, phosphorylation, and hydroxylation) are also key features in regulating DUB activity. Furthermore, regulatory factor in trans (between two molecules) or accessory domain *in cis* (within a molecule) exerts a boosting effect on the catalytic activity. The star indicates the active site.

DUBs are a class of proteases responsible for cleaving peptide or isopeptide bonds between linked ubiquitin molecules or between ubiquitin and a modified protein. The diverse linkage types, length, and topology give rise to the complexity of Ub chain architectures, which requires a broad variety of individual DUB specificity.^[^ ^35^ ^]^ DUB–substrate interactions are thought to be weak and transient in nature state, much like most enzyme–substrate interactions. Overall, most members of the USP and UCH subfamilies have nonspecific interactions with the substrate (without a motif). Whereas, substrate recognition by DUBs is also governed by sequences and motifs outside the conserved catalytic domain. For example, Spt‐Ada‐Gcn5‐acetyltransferase (SAGA) deubiquitination module has substrate specificity through motif interactions with the substrate protein. Ubiquitin linkage specificity is acquired so that only one linkage type is identified by Ub‐binding sites to catalyze DUB activity. Many members of OTU, JAMM, Josephin, and MINDY subfamilies have linkage specificity. Most USPs are recruited to substrates for direct deubiquitination regardless of linkage‐type or architecture.^[^ ^36^, ^37^ ^]^ Exceptionally, a subset of the USPs, including CYLD and USP30, prefers to Lys63‐ and Met1‐linkage.^[^ ^38^, ^39^, ^40^ ^]^ In contrast, a handful of DUBs carry out the function by targeting specific chain types. JAMM subfamily members such as AMSH, AMSH‐LP, BRCC, and POH1 are generally Lys63 specific; and this is the first confirmed DUB family to display linkage specificity.^[^ ^41^, ^42^ ^]^ In addition, numerous OTU DUBs are also linkage‐specific,^[^ ^43^ ^]^ such as the Lys48 linkage specificity of OTUB1,^[^ ^44^ ^]^ the Lys11 linkage specificity of Cezanne (OTUD7B),^[^ ^45^ ^]^ the Met1 linkage specificity of OTULIN,^[^ ^46^ ^]^ and the Lys29 and Lys33 linkages specificity of TRABID.^[^ ^47^ ^]^ The ZUP1 family, with ZUP1 itself as its sole human member, is specific for Lys63‐linkage conferred by multiple UBDs and is associated with genome stability.^[^ ^30^, ^48^, ^49^, ^50^ ^]^ Whereas, each MINDY family member exhibits the specificity for Lys48‐linkage, strongly suggesting this family plays a vital role in protein turnover.^[^ ^28^ ^]^ In addition to direct recognition, some DUBs recognize targeted proteins with the assistance of adaptors or scaffolds. For instance, LINC00857 acts as a protein scaffold by binding to both FOXM1 and OTUB1, thereby enhancing their interaction and inhibiting FOXM1 degradation through the ubiquitin‐proteasome pathway.^[^ ^51^ ^]^ The recruitment of CYLD to HOIP is facilitated by the adaptor protein SPATA2, which acts as a bridge between the DUB and HOIP (**Figure** **4**B).^[^ ^52^ ^]^

The length of Ub chains is distinguishable for linkage types and supplies one more variable. Ub chains can be trimmed by DUBs according to endo‐ or exo‐cleavage activity. Endo‐cleavage activity clears the proximal Ub from a substrate and the unattached Ub chains must be further converted into monoubiquitin. In contrast, exo‐cleavage activity directly produces monoubiquitin from the distal Ub, but multiple actions of DUB are required to remove the en bloc polyubiquitin chain (Figure 4C). Where to cleave, endo or exo, depends on the DUB itself, linkage type, and polyubiquitin identification.^[^ ^35^, ^53^ ^]^ With the advances in biochemistry and chemical biology, several methods have been developed to profile DUB linkage preference, such as mutation of ubiquitin, middle‐down mass spectrometry, development of antibodies, etc.^[^ ^54^ ^]^ What is prominent, Ubiquitin Chain Restriction (UbiCRest) was designed to assess the intrinsic linkage‐specificity of DUBs in a short time. Briefly, a panel of artificially generated and specific linkages was incubated with DUB of interesting. Cleavage products were then resolved on SDS‐PAGE gradient gel and visualized by silver stained or western blot analysis.^[^ ^54^ ^]^ Based on UbiCRest analysis of cleavage patterns and Ub linkage products with the treatment of DUB‐specific enzymes in vitro or in cellulo, the appearance of Ub chain composition and architecture on the substrates can be partly outlined.^[^ ^43^, ^54^ ^]^ Although significant structural information has been disclosed. Predicting the linkage or substrate specificity of DUBs remains challenging.

### 2.3 The Regulation of DUBs’ Enzymatic Activity

Catalytic activity of DUBs is directly governed by a variety of manners, including substrate‐assisted catalysis, allosteric regulation, and PTMs (Figure 4D).^[^ ^17^ ^]^ In some instances, substrate binding directly contributes to enzyme activation, enabling accurate control of specificity in deubiquitination. OTULIN catalytic activity is modulated by a substrate‐assisted mechanism.^[^ ^46^ ^]^ It is unable to trim the Lys63‐linkage; however, its enzymatic activity is directly activated through preferential binding of Met1‐linked ubiquitin.^[^ ^46^, ^55^ ^]^ Likewise, Cezanne undergoes dramatic conformational rearrangements upon interacting with the appropriate polyubiquitin which impairs its turnover and defines its linkage specificity.^[^ ^45^ ^]^ In addition, allostery plays a vital role in adjusting the activity of DUBs. One example is that USP1 binds to USP1‐associated factor 1 (UAF‐1) and converts its ineffective state into an active state through conformational changes.^[^ ^56^ ^]^ Meanwhile, USP7 catalytic activity is controlled by the interaction of internal domains with herpesvirus‐associated USP (HAUSP).^[^ ^57^ ^]^ The last two of five HAUSP ubiquitin‐like domains (HUBLs) and a C‐terminal peptide (CTP) enhance USP7 activity 100‐fold by re‐binding to the so‐called switching loop in the USP domain.^[^ ^57^ ^]^

PTMs also play a crucial role in adjusting catalytic DUB activity in addition to the above‐mentioned PTMs regulation of DUBs localization. Crosstalk between PTMs and the Ub system mainly includes phosphorylation, ubiquitination, SUMOylation, and oxidation.^[^ ^35^, ^58^ ^]^ Phosphorylation can positively or negatively regulate DUB activity. For instance, USP8 is inhibited by 14‐3‐3 proteins in a phosphorylation‐dependent manner, and dephosphorylation in the cell cycle M phase increases USP8 activity.^[^ ^59^ ^]^ Mutations associated with binding motifs in 14‐3‐3 also improve USP8 activity, leading to upregulation of the epidermal growth factor (EGF) receptor signaling pathway which causes Cushing's disease.^[^ ^60^ ^]^ CYLD activity is also controlled by phosphorylation in positive and negative ways. Although IKKγ^[^ ^61^ ^]^ and IKKε^[^ ^62^ ^]^ of the IκB kinase (IKK) family modulate inhibitory phosphorylation, IKKβ phosphorylation activates CYLD.^[^ ^63^ ^]^ Coincidentally, A20 modification by IKKβ^[^ ^64^ ^]^ enhances A20 activity involving Lys63‐linked chains.^[^ ^65^ ^]^ However, the detailed mechanisms of CYLD or A20 regulation by phosphorylation remain unclear, and it is worth noting that the phosphorylation sites are not in their respective catalytic domains. Elucidating the protein interactome of DUBs helps to optimize the fine regulation mechanism of phosphorylation modification in DUB activity and offers novel lights for therapeutic targeting via disrupting this interaction. DUBs can also be ubiquitinated in complexes with E3 ligases. For example, USP30 is ubiquitinated by Parkin in its zinc fingers subdomain, which may impair catalytic activity.^[^ ^66^ ^]^ In contrast, monoubiquitinylation of the ATXN3 Josephin domain boosts enzymatic activity by initiating a conformational change.^[^ ^67^ ^]^ In addition, poly‐SUMOylation enables OTUB2 to deubiquitinate Yes‐associated protein (YAP) and transcriptional coactivator with a PDZ‐binding motif (TAZ).^[^ ^68^ ^]^ Moreover, most DUBs are cysteine proteases harboring a reactive cysteine residue that is vulnerable to be oxidated by reactive oxygen species (ROS). Oxidation attenuates the activity of USP‐, OUT‐, and UCH family members and it may be reversible.^[^ ^69^, ^70^, ^71^ ^]^ Interestingly, the inhibitory or activating modulation of DUBs activity depicts the fascinating intricacy of post‐translational adjustment.

Furthermore, the regulatory factor in trans (between two molecules) or accessory domain *in cis* (within a molecule) exerts a boosting effect on the catalytic activity of individual DUB through allosteric regulation or promoting the interaction with protein substrates. This endows divergent members of multiple DUB families with miscellaneous regulatory mechanisms and expands the broad range of DUBs regulation in various cellular processes.^[^ ^35^ ^]^

Overall, these aspects coordinately modulate the specificity and catalytic activity of DUBs, forming dynamic and diverse interactions between DUBs and protein substrates. Diverse mechanisms of DUB regulation allow for finely tuned functions that guarantees an appropriate response to the external and internal situation.

### 2.4 The Abundance of DUBs

In theory, the simplest mechanism to regulate the activity and consequent biological function of a given DUB is regulating its intracellular abundance, which can be controlled by transcription, translation, and degradation. The copy number of DUBs lies on several orders of magnitude from the low hundreds to hundreds of thousands estimated by mass spectrometry datasets.^[^ ^72^ ^]^ Some high‐copy number DUBs are required for the maintenance of basic cellular functions, which are analogous to housekeeping genes. In mammalian cells, USP14, 26S proteasome non‐ATPase regulatory subunit 14 (PSMD14), and UCHL5 are associated with the 19S particle lid of proteasome to coordinately ensure the essential preprocessing of protein degradation and maintain sufficient levels of free Ub.^[^ ^73^ ^]^ USP14 and UCHL5 trim ubiquitin chains from the distal end, while PSMD14 is responsible for removing whole poly‐UB chain from target proteins degraded by 20S proteasome core particle.^[^ ^74^, ^75^ ^]^ Loss of PSMD14 activity in purified proteasomes prevents protein degradation.^[^ ^53^ ^]^ Two inactive‐ or pseudo‐DUBs, USP39 and PRPF8, have a central role in the mature spliceosome assembly and pre‐mRNA splicing as components of the spliceosome complex.^[^ ^76^, ^77^ ^]^ In addition, some of the other DUBs, such as USP7, USP8, USP36 are essential for cell survival.^[^ ^78^, ^79^, ^80^ ^]^

Notably, lower abundance DUBs can be activated in a stimulation‐dependent manner and exert a particular regulatory effect. For instance, OTU family member A20 (also called TNFAIP3) is hardly expressed in unstimulated cells, but its expression is induced by tumor necrosis factor (TNF) and substantially upregulated upon TLR4‐mediated nuclear factor κB (NF‐κB) activation.^[^ ^81^, ^82^ ^]^ A20 also acts as a negative feedback regulator to inhibit NF‐κB activation, thereby avoiding the overproduction of inflammatory factors. Moreover, A20 protein levels are controlled by MALT1, which hydrolyzes A20 between the N‐terminal catalytic OTU domain and the C‐terminal UBD, thus jeopardizing its function.^[^ ^83^ ^]^ Similarly, cylindromatosis (CYLD) is cleaved and destabilized by MALT1.^[^ ^84^, ^85^, ^86^ ^]^ These examples highlight that DUB abundance can be regulated by non‐specific and situation‐dependent ways to impact cellular function. Individual DUB performs specific effects under certain stimuli and different DUBs can cooperate or antagonize each other to maintain the homeostasis of cellular functions.

### 2.5 The Localization of DUBs

DUBs’ localization can be governed in various ways. Above all, PTMs are a convenient way for cells to affect DUB functional localization.^[^ ^35^ ^]^ Phosphorylation catalyzed by casein kinase 2 is essential for nuclear localization of OTUB1 and ATXN3 (a member of MJD family), which exerts a critical effect in regulating cellular metabolism during hypoxic stress.^[^ ^87^ ^]^ Similarly, USP10 can be phosphorylated by ataxia‐telangiectasia mutated kinase (ATM) and translocate to the nucleus, which interacts with p53 and inhibits p53 nuclear export and degradation; thereby suppressing tumor cell growth.^[^ ^88^, ^89^, ^90^ ^]^ On the contrary, phosphorylation by protein kinase B (PKB/AKT) excludes USP4 from the nucleus, allowing it to reach the plasma membrane and deubiquitinate transforming growth factor‐β (TGF‐β) receptor I (TβRI). This can stabilize the TβR‐I at the plasma membrane and boost TGF‐β signaling, thereby promoting tumorigenesis.^[^ ^91^ ^]^

Moreover, localization is also controlled by altering DUB interactomes. OTUB1 hydroxylation by factor inhibiting HIF (FIH) changes the OTUB1 interactome and substrates.^[^ ^87^ ^]^ Ubiquitination of USP4 regulates its interactome and its roles in DNA damage response,^[^ ^92^ ^]^ whereas binding of phosphorylated OTULIN prevents its interaction with linear Ub chain assembly complex (LUBAC).^[^ ^93^, ^94^ ^]^ Numerous DUBs are produced as multiple splice variants which may localize to different compartments and have distinct half‐lives.^[^ ^53^ ^]^ USP35 has one form which localizes to the ER and lipid droplets and other forms are targeted to the cytosol. A short form of USP35 is linked to the mitochondria, but this variant lacks an intact catalytic domain.^[^ ^95^, ^96^ ^]^

Thus, DUBs localization not only depends on the signal peptide of DUB itself, but also can be affected by PTMs and DUB interactome. This reveals that domains beyond DUBs’ catalytic sites play a pivotal role in regulating their functions.

## 3 The Roles of DUBs in Cancer

**Figure 5:**
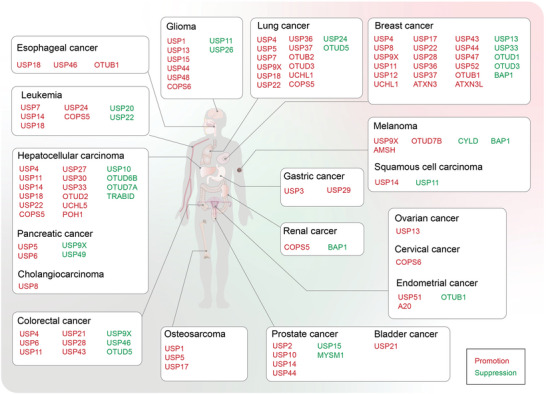
Overview of Janus‐faced roles of DUBs in cancers. DUBs exhibit dual roles in the pathogenesis of diverse cancers, including glioma, lung cancer, breast cancer, melanoma, ovarian cancer, cervical cancer, endometrial cancer, gastric cancer, renal cancer, prostate cancer, bladder cancer, osteosarcoma, colorectal carcinoma hepatocellular carcinoma pancreatic adenocarcinoma cholangiocarcinoma leukemia and esophageal squamous cell carcinoma, affecting multiple organs within the human body. DUBs exhibiting tumor‐promoting function are visually highlighted by red, while those demonstrating tumor‐suppressing function are visually indicated by green.

An increasing number of studies show that DUBs play oncogenic or suppressive functions at multidimensional levels in cancer, which are listed in **Table** **1** and summarized in **Figure** **5**. The diverse functions of DUBs mainly act on the instability, conformational changes, and signal transduction of substrates. Here, the multiple roles of DUBs in cancer are elucidated as the following aspects: proteasomal degradation, DNA repair, apoptosis, and metastasis.

**Table 1:** The roles of DUBs in cancers.

DUB	Roles	Target	Function	Consequences
USP1	Promotor	ULK1, EZH2, ID1/2/3, CHK1	Stabilization	Promote proliferation, metastasis, cancer cell stemness, and regulate DDR^[^ ^117^, ^118^, ^282^, ^283^ ^]^
USP2	Promotor	CCND1, CCNA1, FAS, MDM2, MDM4, TβRI	Stabilization	Promote proliferation and metastasis, evade apoptosis^[^ ^284^, ^285^, ^286^, ^287^, ^288^ ^]^
USP3	Promotor	SUZ12	Stabilization	Boost metastasis^[^ ^289^ ^]^
	Suppressor	p53	Stabilization	Inhibit proliferation^[^ ^290^ ^]^
USP4	Promotor	HDAC2, ARF‐BP1, TβRI, Twist1, β‐catenin, PRL‐3, CYPA,	Stabilization	Promote proliferation and metastasis, decrease sensitivity to chemotherapy^[^ ^91^, ^144^, ^147^, ^291^, ^292^, ^293^ ^]^
USP5	Promotor	c‐Maf, Slug, β‐catenin	Stabilization	Evade apoptosis, promote metastasis^[^ ^294^, ^295^, ^296^ ^]^
USP6	Promotor	c‐Jun, Fzd, JAK1	Stabilization	Promote metastasis and tumor growth^[^ ^297^, ^298^, ^299^ ^]^
USP7	Promotor	CDC25A, N‐Myc, Ki‐67, HIF‐1α, MDM2, MDM4, PHF8, MDC1, β‐catenin, LSD1, NOTCH1, HDM2	Stabilization	Antagonize p53‐involved tumor suppression regulations, Promoting tumor metastasis, decrease drug sensitivity, regulate DDR^[^ ^135^, ^300^, ^301^, ^302^, ^303^, ^304^, ^305^, ^306^, ^307^, ^308^, ^309^, ^310^, ^311^, ^312^, ^313^, ^314^, ^315^, ^316^, ^317^ ^]^
	Suppressor	PD‐L1	Stabilization	Anti‐tumor immune response^[^ ^257^ ^]^
USP8	Promotor	Cx43, AKT, TβRII, PD‐L1	Stabilization	Promote proliferation and metastasis, inhibit immonotherapy^[^ ^318^, ^319^ ^]^
USP9X	Promotor	pVHL, CEP131, TRB3, SMAD4, SMURF1, TDRD3, β‐catenin	Stabilization	Promoting proliferation and metastasis, evade apoptosis^[^ ^320^, ^321^, ^322^, ^323^, ^324^, ^325^, ^326^, ^327^, ^328^, ^329^, ^330^ ^]^
	Suppressor	YAP1, TIK, MCL1, XIAP, Ets‐1, IRS‐2, CLASPIN, FBW7, AMOT, ITCH, LATS2, CLASPIN	Stabilization or altered activity	Maintain DNA replication fork stability and inhibit proliferation^[^ ^127^, ^321^, ^324^, ^331^, ^332^ ^]^
USP10	Promotor	SMAD4, Slug, TOP2α, FLT3, G3BP2	Stabilization or altered activity	Promote tumorigenesis and metastasis^[^ ^333^, ^334^, ^335^, ^336^ ^]^
	Suppressor	MSH2, p53, SIRT6, p14ARF, AMPKα, PTEN, MSH2	Stabilization	Suppress tumor progression and increase apoptosis, regulate cellular sensitivity to DNA damage^[^ ^88^, ^337^, ^338^, ^339^ ^]^
USP11	Promotor	XIAP, cIAP2, RAE1, PPP1CA, NF90, eIF4B, TβRII	Stabilization	Promote proliferation and metastasis, evade apoptosis^[^ ^340^, ^341^, ^342^, ^343^, ^344^, ^345^, ^346^, ^347^ ^]^
	Suppressor	VGLL4, ARID1A, PTEN, Mgl‐1, p21, PML, BRCA2	Stabilization or altered activity	Inhibit tumorigenesis and metastasis, regulate DDR^[^ ^123^, ^124^, ^348^, ^349^, ^350^, ^351^, ^352^, ^353^, ^354^ ^]^
USP12	Promotor	AR, MDK, MDM2	Stabilization	Maintain malignant traits and promote angiogenesis^[^ ^355^, ^356^, ^357^ ^]^
USP13	Promotor	MCL1, c‐Myc, MITF RAP80, ACLY, OGDH	Stabilization	Promote proliferation and metabolism, chemotherapy resistance^[^ ^358^, ^359^, ^360^, ^361^, ^362^ ^]^
	Suppressor	PTEN, RAP80‐BRCA1	Stabilization	Anti‐tumor and regulate DDR^[^ ^359^, ^363^ ^]^
USP14	Promotor	PI3K, Dvl, Vimentin, Aurora B, AR	Stabilization	Promote proliferation and metastasis, prevent apoptosis and regulate DDR^[^ ^103^, ^105^, ^364^, ^365^, ^366^, ^367^ ^]^
USP15	Promotor	TOP2α, MDM2, TβRI, HBx	Stabilization or altered activity	Promote metastasis and inhibit anti‐tumor immunoresponses^[^ ^368^, ^369^, ^370^, ^371^ ^]^
	Suppressor	Keap1, IRS2, p53	Stabilization or altered activity	Inhibit tumorigenesis and decrease chemo‐resistance^[^ ^372^, ^373^, ^374^ ^]^
USP17	Promotor	CDC25A, Geminin, HAS2, Slug, Snail, Twist, SMAD4, BRD4	Stabilization	Promote tumorigenesis and metastasis, prevent apoptosis^[^ ^166^, ^311^, ^375^, ^376^, ^377^, ^378^, ^379^ ^]^
USP18	Promotor	ZEB1, RARα, BCL2L1, KRAS	Stabilization	Promote tumorigenesis and metastasis, prevent apoptosis^[^ ^380^, ^381^, ^382^, ^383^ ^]^
USP19	Suppressor	HDAC1/2	Altered activity	Maintain genomic stability^[^ ^384^ ^]^
USP20	Promotor	β‐catenin	Stabilization	Promote tumorigenesis^[^ ^385^ ^]^
	Suppressor	TRAF6, Tax, CLASPIN	Stabilization or altered activity	Inhibit tumorigenesis^[^ ^386^, ^387^ ^]^
USP21	Promotor	EZH2, BRCA2, MEK2, PD‐L1	Stabilization	Promote tumorigenesis, metastasis, immune escape^[^ ^265^, ^388^, ^389^, ^390^, ^391^ ^]^
	Suppressor	Fra‐1, MARK1	Stabilization	Anti‐tumor^[^ ^392^ ^]^
USP22	Promotor	CCNB1, CCND1, c‐Myc, FBP1, BMI1, COX‐2, EGFR, H2A, KDM1A, SIRT1, PD‐L1	Altered activity or stabilization	Promote proliferation, metastasis and cancer stemness, drug resistance, reduce sensitivity to immune therapy, regulateγH2AX‐mediated DDR^[^ ^265^, ^359^, ^393^, ^394^, ^395^, ^396^, ^397^, ^398^, ^399^, ^400^, ^401^ ^]^
	Suppressor	PU.1	Stabilization	Anti‐differentiation^[^ ^402^ ^]^
USP24	Promotor	β‐TrCP, MCL‐1	Stabilization	Promote cancer malignancy^[^ ^403^, ^404^ ^]^
	Suppressor	Bax, E2F4, p300, Securin	Stabilization	Promote apoptosis^[^ ^405^ ^]^
USP26	Promotor	AR, Snail	Altered activity or Stabilization	Promote proliferation and metastasis^[^ ^406^, ^407^ ^]^
	Suppressor	SMAD7	Stabilization	Inhibit metastasis^[^ ^406^ ^]^
USP27	Promotor	Cyclin E	Stabilization	Pro‐tumor effects^[^ ^408^ ^]^
USP28	Promotor	c‐Myc, Fbw7	Stabilization	Promote tumorigenesis and metastasis^[^ ^409^, ^410^, ^411^, ^412^, ^413^ ^]^
	Suppressor	LIN28A, LSD1, c‐Jun, NICD1, p53	Stabilization	Arrest cell cycle^[^ ^414^ ^]^
USP29	Promotor	Claspin, Snail	Stabilization	Promote tumorigenesis and metastasis^[^ ^415^, ^416^ ^]^
	Suppressor	p53	Stabilization	Inhibit proliferation^[^ ^417^ ^]^
USP30	Promotor	TOM20, DRP1	Stabilization	Promote migration, evade apoptosis^[^ ^418^, ^419^ ^]^
USP33	Promotor	SP1	Stabilization	Pro‐tumor effects^[^ ^420^, ^421^ ^]^
	Suppressor	PPM1A, Robo1, β‐arrestin2	Altered activity or Stabilization	Inhibiting tumor metastasis^[^ ^422^, ^423^, ^424^ ^]^
USP35	Suppressor	ABIN‐2	Stabilization	Inhibit proliferation^[^ ^425^ ^]^
USP36	Promotor	c‐Myc, DHX33, CHD7, H2B	Altered activity or stabilization	Promote tumorigenesis and proliferation^[^ ^426^, ^427^, ^428^, ^429^ ^]^
USP37	Promotor	RARA, Gli1, Snail, 14‐3‐3γ, c‐Myc	Stabilization	Promote proliferation and metastasis^[^ ^430^, ^431^, ^432^, ^433^, ^434^ ^]^
	Suppressor	p27	Stabilization	Inhibit proliferation^[^ ^435^ ^]^
USP42	Suppressor	p53	Stabilization	Inhibit proliferation^[^ ^436^ ^]^
USP43	Promotor	H2B, ZEB1	Altered activity or Stabilization	Promote proliferation and metastasis^[^ ^156^, ^437^ ^]^
USP44	Promotor	H2B, EZH2, Securin	Altered activity or Stabilization	Promote proliferation and metastasis^[^ ^438^, ^439^, ^440^, ^441^ ^]^
USP46	Promotor	CDT2, ENO1	Stabilization	Promote proliferation and metastasis^[^ ^442^, ^443^ ^]^
	Suppressor	PHLPP	Stabilization	Inhibit proliferation^[^ ^444^ ^]^
USP47	Promotor	β‐catenin, Snail	Stabilization	Promote proliferation and metastasis^[^ ^445^, ^446^, ^447^ ^]^
USP48	Promotor	TRAF2, Gli1	Stabilization	Promote tumorigenesis and metastasis^[^ ^448^ ^]^
USP49	Suppressor	p53, FKBP51	Stabilization	Inhibit proliferation and regulate DDR^[^ ^449^, ^450^ ^]^
USP51	Promotor	ZEB1, FAT4	Stabilization	Promote proliferation and metastasis^[^ ^451^, ^452^ ^]^
USP52	Promotor	ASF1A	Stabilization	Drug resistance^[^ ^453^ ^]^
CYLD	Suppressor	Dvl, p53, TRAF2, Bcl‐3, c‐Jun, c‐Fox	Stabilization	Suppress tumorigenesis and metastasis, promote apoptosis, regulate DDR^[^ ^454^, ^455^, ^456^, ^457^, ^458^, ^459^, ^460^ ^]^
OTUB1	Promotor	c‐IAP1, FOXM1, Snail, SLC7A11, PD‐L1, UBC13	Stabilization	Promote tumorigenesis, metastasis and immune escape, regulate DDR^[^ ^131^, ^461^, ^462^, ^463^, ^464^, ^465^ ^]^
	Suppressor	Erα, RPA1, DEPTOR	Stabilization or altered activity	Suppress tumorigenesis^[^ ^466^, ^467^, ^468^ ^]^
OTUB2	Promotor	YAP/TAZ, U2AF2	Stabilization	Promote metastasis^[^ ^68^, ^469^ ^]^
OTUD1	Suppressor	YAP, p53, SMAD7	Stabilization or altered activity	Suppress tumorigenesis cancer stemness and metastasis^[^ ^470^, ^471^, ^472^ ^]^
OTUD2	Promotor	ITCH	Stabilization	Promote proliferation^[^ ^473^ ^]^
OTUD3	Promotor	GRP78	Stabilization	Promote tumorigenesis^[^ ^474^ ^]^
	Suppressor	PTEN, p53	Stabilization	Inhibit proliferation and induce apoptosis^[^ ^475^, ^476^ ^]^
OTUD5	Suppressor	p53, PDCD5, UBR5	Stabilization	Promote apoptosis and drug sensitivity^[^ ^477^, ^478^, ^479^ ^]^
OTUD6B	Suppressor	pVHL	Stabilization	Inhibit metastasis^[^ ^480^ ^]^
OTUD7A	Suppressor	TRAF6	Altered activity	Inhibit metastasis^[^ ^481^ ^]^
OTUD7B	Promotor	GβL, Aurora A, Cyclin B, EGFR	Stabilization or altered activity	Promote turiogenesis, proliferation, angiogenesis^[^ ^482^, ^483^, ^484^, ^485^ ^]^
A20	Promotor	ERα	Stabilization	Promote proliferation^[^ ^486^ ^]^
TRABID	Promotor	EZH2	Stabilization	Promote proliferation^[^ ^487^ ^]^
	Suppressor	Twist1	Altered activity	Inhibit metastasis^[^ ^488^ ^]^
UCHL1	Promotor	HIF‐1α, TβRI, SMAD2	Stabilization	Pro‐metastasis^[^ ^169^, ^489^ ^]^
	Suppressor	NOXA, p53	Stabilization	Inhibit proliferation, induce chemosensitivity^[^ ^141^, ^490^ ^]^
UCHL3	Promotor	TDP1	Stabilization	Drug resistance^[^ ^491^ ^]^
UCHL5	Promotor	TβRI, PRP19	Stabilization	Promote tumorigenesis and metastasis^[^ ^107^, ^492^ ^]^
BAP1	Suppressor	H2A, γ‐tubulin, Ino80, IP3R3, MCRS1	Altered activity or Stabilization	Maintain chromosomal stability, inducecell apoptosis^[^ ^493^, ^494^, ^495^, ^496^, ^497^ ^]^
ATXN3	Promotor	KLF4	Stabilization	Promote proliferation^[^ ^498^ ^]^
	Suppressor	p53	Stabilization	Induce apoptosis^[^ ^499^ ^]^
ATXN3L	Promotor	KLF5	Stabilization	Promote proliferation^[^ ^500^ ^]^
POH1	Promotor	E2F1	Stabilization	Promote proliferation^[^ ^98^ ^]^
COPS5	Promotor	PD‐L1, HK2, Trx, Snail, Survivin, FOXM1, ZEB1	Stabilization	Reduce sensitivity to immune therapy, promote proliferation and metastasis^[^ ^263^, ^501^, ^502^, ^503^, ^504^, ^505^ ^]^
COPS6	Promotor	CHIP, CTSL, PD‐L1	Altered activity	Promote immune escape, promote proliferation and metastasis^[^ ^506^, ^507^, ^508^ ^]^
AMSH	Promotor	Slug	Stabilization	Promote metastasis^[^ ^509^ ^]^
BRCC36	Promotor	NuMA	Altered activity	Pro‐proliferation^[^ ^510^ ^]^
MYSM1	Suppressor	H2A	Altered activity	Anti‐tumor effects^[^ ^511^ ^]^

### 3.1 DUBs in Tumorigenesis

Proteasomal degradation plays a crucial role in governing the turnover and function of intracellular proteins involving in cancer cell growth and death. Taken the aforementioned proteasome‐related DUBs, USP14, PSMD14, and UCHL5, for instance. The levels of PSMD14 are negatively correlated with the survival of patients with multiple myeloma and its depletion affects the proliferation of multiple myeloma cells.^[^ ^97^ ^]^ Elevated nuclear PSMD14 is seen in hepatocellular carcinomas and promotes tumour growth.^[^ ^98^ ^]^ Furthermore, PSMD14 has been identified to control the ubiquitination and stability of the oncogene receptor tyrosine kinase ERBB2.^[^ ^99^ ^]^ PSMD14 deubiquitinates Ub‐chain at the double‐strand break (DSB) sites to promote the recruitment of RAS associated with diabetes protein 51 (RAD51), which enhances the cellular responses to DSB and undergoes DSB signaling and repair by homologous recombination (HR).^[^ ^100^ ^]^ PSMD14 depletion sensitizes and kills cancer cells with a strong reliance on HR.^[^ ^100^ ^]^

USP14 expression is connected with colorectal cancer, intrahepatic bile duct cancer, lung cancer, and ovarian cancer.^[^ ^101^, ^102^ ^]^ High USP14 expression correlates with poor prognosis in non‐small‐cell lung carcinoma (NSCLC).^[^ ^103^ ^]^ Up‐regulation of USP14 in cisplatin‐resistance ovarian cancer contributes to cancer cell proliferation by stabilizing oncoprotein B‐cell lymphoma 6 (BCL6).^[^ ^104^ ^]^ Moreover, high USP14 expression guards Aurora‐B (an anti‐apoptotic protein) against degradation and inhibits cell apoptosis induced by chemotherapeutic drugs in leukemic cells.^[^ ^105^ ^]^ Similarly, UCHL5 is overexpressed in epithelial ovarian cancer, which is closely associated with advanced tumor progression and poor prognosis.^[^ ^106^ ^]^ UCHL5 is also upregulated in hepatocellular carcinoma, and promote cells migration and invasion by deubiquitinating pre‐mRNA‐processing factor 19 (PRP19).^[^ ^107^ ^]^ Unlike PSMD14, UCHL5 and USP14 are not fundamental constituents of the proteasome. Interestingly, depletion of either UCHL5 or USP14 alone has no measurable effect on cell viability and structure or proteolytic capacity of the proteasome, but does facilitate cellular protein degradation. In comparison, depletion of both of these DUBs reduces protein degradation, indicating that there are overlapping functions between them.^[^ ^108^ ^]^

### 3.2 DUBs in Tumor DNA Damage Repair

**Figure 6:**
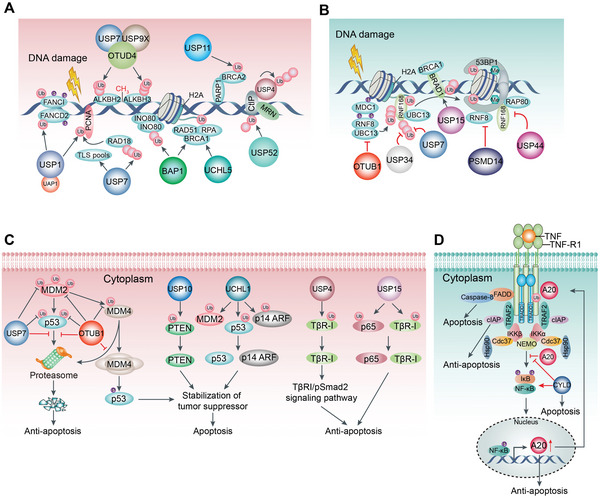
DUBs play multi‐faced roles in tumor‐associated DNA repair and apoptosis. A) Replication‐associated repair is regulated by DUBs. USP1 promotes multiple rounds of repair in the S phase by modulating the turnover of FANCD2‐FANCI monoubiquitination at the damaged sites. Similarly, PCNA is deubiquitinated by USP1 to govern the error‐prone translesion synthesis repair pathway. USP7 indirectly coordinates PCNA deubiquitination. OTUD4 acts as a scaffold to form a complex with USP7 and USP9X which interacts with ALKBH2 and ALKBH3 to suppress their ubiquitination and degradation. ALKBH2 and ALKBH3 preferentially remove methyl groups from single‐stranded and double‐stranded DNA and reverse DNA alkylation. In addition, USP9X upholds the stability of the DNA replication fork and the response of DNA damage checkpoint by modulating the protein claspin in the S phase. BAP1 promotes HR repair by recruiting BRCA1, RAD51, and RPA to DSB sites and reduces ubiquitinated forms of H2A and H2AX. USP11 forms a complex with BRCA2 that boosts HR in DSB sites and limits PARP1 activity. DUBs govern Ub‐dependent signaling, contributing to DNA repair. USP4 autodeubiquitylation regulates CtIP recruitment to sites of DNA damage. USP52 deubiquitinates CtIP, thereby promoting and facilitating the phosphorylation and activation of CtIP for DNA repair. B) Double‐strand breaks (DSBs) promote ATM kinase to phosphorylate histone H2A and MDC1, a mediator of DNA damage checkpoint protein 1. This leads to the recruitment of E3 ligase RNF8 conjugated with E2 enzyme UBC13 that induces the production of Lys63‐linked Ub chains on scaffold proteins (such as RNF168) in response to DNA damage. Several DUBs counteract this ubiquitination in diverse manners including OTUB1, USP34, and USP7, although some are functionally redundant. Additionally, oligomerized 53BP1 is stably recruited by RNF168‐ and RNF8‐regulated chromatin ubiquitination, which is inhibited by PSMD14 and USP44. USP15 deubiquitinates BARD1, and promotes BARD1‐HP1γ interaction, resulting in BRCA1/BARD1 retention at DSBs. C) The role of DUBs in regulating apoptosis. DUBs increase or decrease apoptosis by inhibiting proteasomal degradation of pro‐apoptotic or anti‐apoptotic proteins through deubiquitination. USP7, OTUB1, UCHL1, and USP10 promote apoptosis by enhancing the stability of tumor suppressors such as p53, PTEN, and p14 ARF. Conversely, the oncogene of TβR‐I and p65 are stabilized by USP4 and USP15, which exert an anti‐apoptotic effect. D) Moreover, A20 is part of a negative feedback mechanism inhibiting continuous NF‐κB activation during single TNF stimulation, wherein A20 expression is constitutively activated and prevents cells from apoptosis through stabilizing the Ub network involved in the TNFR1 signaling complex. However, the DUB CYLD antagonizes this process.

Dysregulation or loss of DNA repair and DNA damage response pathways (DDR) is one hallmark of cancer due to DSBs; this lethal damaging event must be repaired before cell division occurs.^[^ ^109^, ^110^, ^111^ ^]^ Ubiquitination and deubiquitination closely participate in DNA repair and DDR.^[^ ^112^, ^113^ ^]^ DUBs perform a multifaceted role in DNA repair during tumorigenesis (**Figure** **6**A and **Table** **2**). USP1 regulates Fanconi anemia group D2 protein (FANCD2) ubiquitination which plays a vital role in the Fanconi anemia pathway of DNA crosslink repair.^[^ ^114^, ^115^ ^]^ USP1 depletion increases voluntary monoubiquitination of FANCD2 and impairs accumulation of the Fanconi anemia core complex at DNA damage sites in a cell cycle‐dependent manner.^[^ ^115^ ^]^ USP1 promotes multiple rounds of repair in the S phase by modulating the turnover of FANCD2‐ Fanconi anemia group I protein (FANCI) monoubiquitination at the damaged sites. Similarly, USP1 deubiquitinated the proliferating cell nuclear antigen (PCNA) to govern the error‐prone translesion synthesis repair pathway.^[^ ^116^ ^]^ In addition, USP1 activities are involved in regulating a feedback loop to inhibit DDR checkpoint kinase 1(CHK1) activity,^[^ ^117^ ^]^ thereby regulating cellular differentiation in osteosarcoma cells via deubiquitination, and impairing the stability of DNA‐binding protein inhibitors.^[^ ^118^ ^]^ USP4 can directly regulate DNA end resection. USP4 autodeubiquitylation regulates CtIP recruitment to sites of DNA damage.^[^ ^119^, ^120^, ^121^ ^]^ USP52 can directly interact with CtIP and deubiquitinate it, thereby promoting and facilitate the phosphorylation and activation of CtIP for DNA repair.^[^ ^122^ ^]^ USP11 is also a DNA repair‐associated DUB that forms a complex with breast cancer type 2 susceptibility protein (BRCA2), which boosts HR in DSB sites and plays a tumor suppressor role in DDR.^[^ ^123^ ^]^ Inhibiting USP11 activity sensitizes cancer cells to olaparid which limits the activity of DDR enzyme poly(ADP‐ribose) polymerase 1 (PARP1).^[^ ^124^ ^]^ USP15 deubiquitinates BARD1, and promotes BARD1‐HP1γ interaction, resulting in BRCA1/BARD1 retention at DSBs. Cancer‐associated USP15 mutations increase PARP inhibitor sensitivity in cancer cells.^[^ ^125^ ^]^

**Table 2:** The function of DUBs in DNA damage response.

DUB	Target	Process	Rationale
USP1	FANCI	Homologous recombination (HR)	Efficient foci formation at sites of DNA damge^[^ ^274^ ^]^
			Promote homologous recombination^[^ ^512^ ^]^
	PCNA	Translesion synthesis (TLS)	Promote recruitment of TLS polymerases^[^ ^116^ ^]^
USP4	USP4 itself	HR	USP4 autodeubiquitylation regulates CtIP recruitment to sites of DNA damage^[^ ^119^, ^120^, ^121^ ^]^
USP7	PCNA	HR	Regulate DNA polymerase η stability^[^ ^513^ ^]^
	Rad18	TLS	Maintain Rad18 stability and DNA damage tolerance^[^ ^514^ ^]^
	RNF168	TLS	Stabilize RNF168 and regulate BRCA1 recruitment at DSB sites^[^ ^515^ ^]^
	RNF169	TLS	Support the nuclear function of DSB response protein^[^ ^516^ ^]^
	ERCC6	Nucleotide‐excision repair (NER)	Regulate the stability of ERCC6 for transcription‐coupled NER^[^ ^517^ ^]^
	XPC	NER	Regulate NER via deubiquitinating XPC^[^ ^518^ ^]^
USP7S	Mule	Base excision repair (BER)	Prevent Mule self‐ubiquitination and regulate DNA damage and repair^[^ ^519^ ^]^
USP9X	MCL1	HR	Stabilize MCL‐1 and promote homologous recombination^[^ ^329^, ^520^ ^]^
	ALKBH2	BER	Promote alkylation damage resistance^[^ ^126^ ^]^
USP11	XPC	NER	Maintain NER capacity via deubiquitinating XPC^[^ ^350^ ^]^
	H2AX	HR	Maintain the proper status of ubiquitylation γH2AX to repair DSB^[^ ^521^ ^]^
USP15	BARD1	BER	USP15 deubiquitinates BARD1, and promotes BARD1‐HP1γ interaction, resulting in BRCA1/BARD1 retention at DSBs^[^ ^125^ ^]^
USP24	DDB2	NER	Regulate DDB2 stability and homologous recombination repair^[^ ^124^, ^522^ ^]^
USP34	RNF168	HR	Promote ubiquitin signaling at DNA double‐strand breaks^[^ ^523^ ^]^
USP45	ERCC1	NER	Promote translocation of ERCC1 to foci of DNA damage^[^ ^524^ ^]^
USP47	Polymerase β	BER	Regulate DNA repair and maintain genome integrity^[^ ^525^ ^]^
USP52	CtIP	HR	Remove the ubiquitination of CtIP to facilitate the phosphorylation and activation of CtIP^[^ ^122^ ^]^
BAP1	BRCA1	HR	Promote DNA repair and cellular recovery from DNA damage^[^ ^526^ ^]^
UCHL5	Ino80	BER	Regulate transcription and DNA repair^[^ ^527^ ^]^
OTUB1	UBC13	HR	Suppress DNA damage response^[^ ^131^ ^]^
OTUB2	L3MBTL1	HR	Fine‐tune DSB speed and regulate DNA repair pathway^[^ ^528^ ^]^
OTUD4	ALKBH2	BER	Promote alkylation damage resistance via stabilization of AlkB homologues^[^ ^126^ ^]^
BRCC36	BRCA1	HR	Limit DNA Break Processing and Repair^[^ ^529^ ^]^

AlkB homolog (ALKBH) 2 and 3 preferentially remove methyl groups from single‐stranded and double‐stranded DNA and reverse DNA alkylation, one of the most common mutagenic events in the cell.^[^ ^126^ ^]^ OTUD4 serves as a scaffold to form a complex with USP7 and USP9X which then interacts with ALKBH2 and ALKBH3 to suppress ubiquitination and degradation.^[^ ^126^ ^]^ USP9X activity can uphold the stability of the DNA replication fork and DDR checkpoint by modulating the protein claspin in the S phase.^[^ ^127^ ^]^ Additionally, USP9X influences radiosensitivity in glioblastoma cells through Myeloid cell leukemia 1 (MCL1)‐dependent or ‐independent mechanisms.^[^ ^128^ ^]^ Breast cancer susceptibility gene 1 (BRCA1) associated protein 1 (BAP1) is a nuclear DUB with a UCH domain. It functions as a tumor suppressor on breast cancer growth.^[^ ^129^ ^]^ BAP1 promotes HR repair by recruiting BRCA1, RAD51, and replication protein A (RPA) to DSB sites and reducing ubiquitinated forms of H2A and γH2AX.^[^ ^130^ ^]^

The above DUBs all play positive regulatory roles in DNA repair; however, several other DUBs respond to DNA repair with opposing effects (Figure 6B and Table 2). OTUB1 is a Lys48 linkage‐specific DUB; however, OTUB1 overexpression inhibits Lys63 chains by interacting with ubiquitin‐charged E2 enzyme UBC13 and disrupting ring finger protein 168 (RNF168)‐mediated polyubiquitination during DSB repair.^[^ ^131^ ^]^ Intriguingly, OTUB1 efficiently limits the DDR mediator/adaptor p53 binding protein 1 (53BP1) foci formation independent of its catalytic activity.^[^ ^131^ ^]^ Moreover, PSMD14 cleaves the Lys63 chains to modulate DSB repair signaling, in addition to disassembling the Lys48 chains of proteasomal substrates.^[^ ^41^ ^]^ PSMD14 depletion enlarges the formation of the nuclear foci for Ub and 53BP1, indicating that the DSB repair signal propagates without DUB, and PSMD14 estranges the role of RNF8 in Lys63 chains formation and nonhomologous end joining (NHEJ) repair.^[^ ^132^ ^]^ In addition, USP44 is an antagonist of RNF168‐regulated Ub conjugates at DSB sites.^[^ ^133^ ^]^ The highly abundant USP44 curbs foci formation of ionizing radiation (IR)‐induced Ub conjugates, RNF168, receptor‐associated protein 80 (RAP80), and 53BP1 at DSB sites through antagonizing IR‐ and RNF168‐mediated H2A ubiquitination.^[^ ^133^ ^]^

These examples reveal that DUBs implement multiple regulatory effects on DNA repair and DDR pathways, including recruitment and stability of core regulators, deubiquitination of nucleosome core particle, and protein interactome in a catalytically independent manner at DNA damage sites. However, DUBs perform a multifaceted role in DNA repair during tumorigenesis, further in‐depth research is urgently needed to determine the detailed and fine‐tuned regulatory mechanisms of DUBs.

### 3.3 DUBs in Tumor Apoptosis

Apoptosis‐mediated cell death serves as a pivotal tumor suppressor in cancer progression. DUBs are demonstrated as a main regulator of apoptosis.^[^ ^134^ ^]^ USP7 can remove Ub‐chain on Mdm2 conjugated by self‐ubiquitination, thus supporting Mdm2‐dependent p53 degradation.^[^ ^135^, ^136^ ^]^ USP7 depletion stimulates MDM2 proteasomal degradation and promotes p53‐mediated apoptosis in tumor cells through stabilizing p53.^[^ ^137^, ^138^ ^]^ In addition, p53 and MDM2 are also regulated by OTUB1, which controls p53 stabilization and activation by disrupting the interaction between p53 and MDM2.^[^ ^139^ ^]^ Moreover, OTUB1 can stabilize murine double minute 4 (MDM4) by curbing MDM4 ubiquitination mediated by MDM2. This promotes MDM4 accumulation in the cytoplasm and mitochondria, p53 phosphorylation, and mitochondrial‐mediated apoptosis.^[^ ^140^ ^]^ UCHL1 forms a complex with p53/p14 ARF/ MDM2 to deubiquitinates p53, and p14 ARF for p53 signaling and apoptosis triggering in nasopharyngeal carcinoma and breast cancer.^[^ ^141^, ^142^ ^]^ USP10 is another DUB with a pro‐apoptosis function; it modulates phosphatase and tensin homolog (PTEN) in lung and breast cancer cell lines.^[^ ^143^ ^]^ Knockdown of USP10 promotes tumor growth, invasion, and metastasis antagonized by PTEN overexpression (Figure 6C).^[^ ^143^ ^]^

Moreover, USP4 can deubiquitinate and stabilize the TβRI, which plays a positive role in liver tumorigenesis.^[^ ^144^ ^]^ Subsequent activation of the TβRI/pSmad2 signaling pathway leads to cell migration and invasion in glioblastoma, breast cancer, liver, and ovarian cancer.^[^ ^145^, ^146^, ^147^ ^]^ In addition, USP15 is up‐regulated in degenerative nucleus pulposus (NP) cells.^[^ ^148^ ^]^ Its overexpression reduces AKT phosphorylation and accelerates cell apoptosis by enhancing the ubiquitination of FK506‐binding protein 5 (FKBP5).^[^ ^148^ ^]^ Furthermore, NF‐κB p65 (RelA) and TβR‐I are potent targets for USP15 through deubiquitination, which plays multiple roles in mediating tumorigenesis in various carcinomas (Figure 6C).^[^ ^149^ ^]^

On the contrary, several DUBs diminish apoptosis by controlling proteasomal degradation of pivotal anti‐apoptotic protein substrates via deubiquitination. A20 has a potent anti‐inflammatory function as part of a negative feedback mechanism by inhibiting NF‐κB activation.^[^ ^150^ ^]^ A20 deficiency increases receptor‐interacting protein kinase 1 (RIPK1) dependent and independent apoptosis in this process. Upon single tumor necrosis factor‐α (TNF‐α) stimulation, A20 expression is then constitutively activated and interacts with the TNF receptor 1 (TNFR1) signaling complex, which prevents apoptosis through stabilizing the Ub network involved in TNFR1 signaling complex.^[^ ^151^ ^]^ Intriguingly, this process is independent of A20's E3 ligase‐ and deubiquitylase activities and can be antagonized by another DUB, CYLD (Figure 6D).^[^ ^152^ ^]^

Whereas, some DUBs play a Janus‐faced role in pro‐/anti‐apoptois of cancer, including UCHL5, USP2, USP4, USP7, USP9X, USP10, and USP14.^[^ ^153^, ^154^ ^]^ The reasons for the opposing roles depend on diverse functions of linkage type for deubiquitination, selective protein substrates associated with oncogenic or tumor suppressive effect, individual interactome, and an ever‐changing microenvironment. It is worth noting that tumor heterogeneity endows extensive differential expression and divergent regulatory roles of DUBs.

### 3.4 DUBs in Tumor Invasion and Metastasis

**Figure 7:**
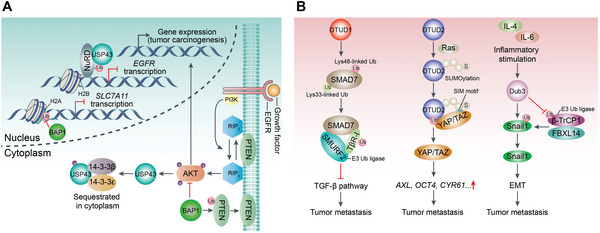
DUBs regulate tumor metastasis. A) USP43 interacts with NuRD to synergistically catalyze H2BK120 deubiquitination and repress EGFR expression which suppresses the activity of the EGFR/PI3K/AKT pathway, as well as the growth and metastasis of cancer. However, USP43 can be phosphorylated by activated AKT, and sequestrated in the cytoplasm to limit USP43 function. Additionally, BAP1 deubiquitinates and stabilizes PTEN (a protein that negatively governs the AKT pathway). Besides, BAP1 deubiquitinates H2A on the *SLC7A11* promoter and represses SLC7A11 expression, which causes ferroptosis to control tumor development and metastasis. B) OTUD1 has dual effects on SMAD7 according to the Ub linkage type. OTUD1 selectively cleaves Lys48‐linked Ub chain from SMAD7 to prevent it from proteasomal degradation which promotes tumor metastasis. Moreover, OTUD1 eliminates Lys33‐linked Ub chain from SMAD7, which can be subsequently bound by SMURF2 (E3 Ub‐protein ligases) to degrade TβRI through ubiquitination, thereby inhibiting the TGF‐β pathway and tumor metastasis. SUMOylated OTUB2 interacts with YAP/TAZ by the SUMO‐interacting motif in YAP and TAZ which promotes the accumulation of YAP and TAZ through deubiquitination and the transcriptional activation of *AXL*, *OCT4*, *CYR61* to increase cell proliferation and metastasis. Dub3 is induced by IL‐4 and IL‐6, and stabilizes Snail1 (a pivotal EMT‐driving transcription factor) through its deubiquitinase activity, and promotes tumor cell migration, invasion, and metastasis.

The leading cause of cancer‐related mortality is metastasis which involves tumor cell migration, extravasation, circulation, immune escape, intravasation into the adjacent tissues, and cell death.^[^ ^155^ ^]^ DUB dysregulation disturbs the level of ubiquitination and function of proteins associated with tumor metastasis which ultimately induces tumor deterioration. For illustration, USP43 is a nuclear‐localized DUB, which is tightly connected with the nucleosome remodeling and deacetylase (NuRD) particle.^[^ ^156^ ^]^ The USP43‐NuRD complex synergistically deubiquitinates H2BK120 and represses a host of genes, including epidermal growth factor receptor (EGFR). USP43 thus robustly suppresses breast cancer growth and metastasis.^[^ ^156^ ^]^ In breast cancer, USP43 can be phosphorylated on Ser29 by activated AKT and sequestrated in the cytoplasm through interacting with the 14‐3‐3β/ε heterodimer.^[^ ^156^ ^]^ Decreased nuclear USP43 leads to EGFR upregulation, which further strengthens the EGFR/PI3K/AKT pathway to accelerate breast cancer progression.^[^ ^156^ ^]^ BAP1 deubiquitinates PTEN and stabilizes it, which allows PTEN to negatively mediate AKT kinase activity. BAP1 inhibition causes the reduction of PTEN protein and enhancement of the AKT signaling pathway which accelerates the malignant transformation and metastasis in prostate cancer.^[^ ^157^ ^]^ Besides, BAP1 deubiquitinates histone 2A (H2A) ubiquitination on the cystine/glutamate transporter (SLC7A11) promoter and represses SLC7A11expression which causes ferroptosis to control tumor development and metastasis.^[^ ^158^, ^159^, ^160^ ^]^ OTUD1 selectively trims the Lys48‐chains from SMAD7 to prevent it from proteasomal degradation in breast cancer (**Figure** **7**A).^[^ ^161^ ^]^ Moreover, OTUD1 eliminates the Lys33‐chains and exposes the PY motif of SMAD7, which can subsequently bind to E3 ligases SMURF2 for TβRI degradation and ubiquitination.^[^ ^162^, ^163^ ^]^ Consequently, OTUD1 acts dual effects on SMAD7 to mitigate TGF‐β signaling and inhibit metastasis.

In contrast, some DUBs promote tumor metastasis (Figure 7B). OTUB2 can be poly‐SUMOylated on lysine 233 and SUMOylated‐OTUB2 can interact with yes‐associated protein (YAP)/transcriptional coactivator with PDZ‐binding motif (TAZ) by the SUMO‐binding motif of YAP and TAZ.^[^ ^68^ ^]^ OTUB2 promotes the accumulation of YAP and TAZ through deubiquitination, and their translocation to the nucleus where they interact with the TEA domain family of transcription factors to activate genes that potentiate cell proliferation and metastasis in a Hippo‐independent manner.^[^ ^68^ ^]^ Dub3 is an early inducible DUB by cytokines, such as IL‐4 and IL‐6.^[^ ^164^ ^]^ In breast cancer, overexpressed Dub3 can stabilize epithelial‐mesenchymal transition (EMT)‐driving transcription factor Snail1^[^ ^165^ ^]^ through its deubiquitylase activity to promote tumor cell migration, invasion, and metastasis.^[^ ^166^ ^]^ Therefore, Dub3 rapidly responds to inflammatory stimulation and exerts its tumor‐promoting effect by stabilizing Snail1.^[^ ^164^, ^166^ ^]^ USP7 interacts with N‐methyltransferase enhancer of zeste homolog 2 (EZH2) to eliminate ubiquitination and improve forkhead box protein A1 (FOXA1) stability, which facilitates the progression of prostate cancer.^[^ ^167^, ^168^ ^]^ Moreover, UCHL1 facilitates TGFβ signaling‐induced breast cancer metastasis by protecting TβRI and SMAD2 from ubiquitination.^[^ ^169^ ^]^

Taken together, the functions of DUBs in cancer can be roughly classified in the following aspects. On the one hand, nuclear‐localized DUBs can directly or indirectly govern the transcription of tumor driver genes through deubiquitylating on histones or forming a complex with cofactors. Additionally, these DUBs also can serve as a scaffold to recruit core regulators for DNA repair. On the other hand, cytosolic‐localized DUBs exert diverse effects by stabilizing classical tumor suppressors or promotors, such as PTEN, TβRI, and p53. This involves the broad crosstalk between EGFR/PI3K/AKT, TGF‐β/SMAD, and NF‐κB signaling. It should be noted that potent inhibitors restraining DUBs activity may exhibit opposite anticancer efficacy in different cancer types.

## 4 The Roles of DUBs in Immunity

Innate immunity is the first line of host defense against invading viral pathogens. It is mainly activated through the recognition of pathogen‐associated molecular patterns (PAMPs) by pattern recognition receptors (PRRs).^[^ ^170^, ^171^ ^]^ There are three classes of PRRs: Toll‐like receptors (TLRs), retinoic acid‐inducible gene I (RIG‐I)‐like receptors (RLRs), and DNA sensor cyclic GMP‐AMP (cGAMP) synthase (cGAS).^[^ ^172^ ^]^ These receptor‐initiated signaling cascades contribute to the production and secretion of pro‐inflammatory cytokines and type I interferon (IFN) to elicit immune responses.

Given that both excessive increase or decrease in PRRs signaling may cause physiological disorders. PRRs signaling is thus tightly controlled at both spatial and temporal levels in a coordinated manner. For instance, spatially, compartmentalization has been recognized as a major safeguard mechanism that prevents PRRs from activation by self‐DNA/RNA, such as restraining cGAS on the plasma membrane through PI(4,5)P_2_ binding^[^ ^173^ ^]^ or in the nucleus via nucleosomes/BAF association.^[^ ^174^ ^]^ In addition, multiple truncated isoforms of MAVS prevent its spontaneous aggregation by interacting with full‐length MAVS and spatially isolating MAVS monomers from each other on the outer mitochondrial membrane.^[^ ^175^ ^]^ MAVS could localize on peroxisomes to induce the rapid interferon‐independent expression of defense factors that provide short‐term protection.^[^ ^176^ ^]^ Temporally, relative long‐term responses of PRRs signaling are transcriptionally regulated.

**Figure 8:**
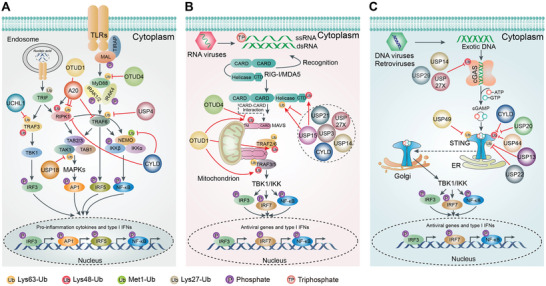
DUBs in innate immune receptor signaling. Innate immunity is activated by signaling cascades associated with pattern recognition receptors (PRRs), including TLRs (A), RLRs (B), and cGAS (C). A) Almost all reported DUBs play a negative regulatory role in TRL signaling, including A20, CYLD, UCHL1, OTUD4, and USP4. These DUBs promote signaling transduction and stabilization of the key mediators in TLR signaling cascades by cleaving the Lys63‐ or Lys48‐linked Ub‐chain from them, such as TRAF6, MyD88, NEMO, TRAF3, and RIP1. This avoids excessive production of pro‐inflammatory cytokines and IFNs. B) Unlike TLR signaling, RLRs can be selectively positively or negatively regulated by DUBs through removal of non‐degradative Lys63‐linked Ub chains or degradative Lys48‐linked Ub chains. CYLD removes Lys63‐linked polyubiquitin chains from RIG‐I, which disrupts the fundamental activation levels and impairs the production of IFN induced by RIG‐I. Additionally, USP3, USP21, USP14, USP27X, and USP15 similarly serve as negative regulators of signaling governed by RIG‐I by removing Lys63‐linked ubiquitination on RIG‐I. However, USP15 also exerts an opposite function through cleaving Lys48‐linked Ub‐chain, which contributes to continuing type I IFN expression. Besides, USP17, USP4, and OTUD4 remove the Lys48‐linked Ub chains from RIG‐I, MDA5, and MAVS, respectively, thereby limiting their proteasomal degradation and facilitating the expression of type I IFN and Antiviral Responses. C) USP14 and USP27X stabilize cGAS by removing the Lys48‐linked Ub‐chain during cGAS‐STING signaling, and promote signaling cascades and type I IFN production. STING can also be stabilized by USP20, CYLD, and USP44 via cleaving Lys48‐linked Ub‐chain. Meanwhile, USP49 removes Lys63‐linked Ub‐chain of STING which leads to decreased interactions between STING and TBK1, and inhibits Antiviral Responses. Moreover, USP13 and USP22 exert a comparable effect on STING and play a negative regulatory role in cGAS‐STING signaling.

PRRs signaling is also fine‐tuned by different PTMs of PRRs and other PRRs signaling components, by which a proper and quick innate‐immune response is triggered under distinct conditions. Ubiquitination widely participates in the innate immune signaling cascades (**Figure** **8**). Notably, non‐degradative Met1‐/Lys33‐/Lys63‐linked Ub‐chain governs the critical upstream event of recruiting recognition receptors and activating numerous signal transduction cascades to resist pathogens and maintain immune homeostasis.^[^ ^17^ ^]^ Ubiquitination of adaptor TRAF3 with Lys33‐linked Ub‐chain serves to connect innate immune signaling to the cellular trafficking apparatus, which crucially ensured temporal and spatial accuracy in response to innate immune signaling.^[^ ^177^ ^]^ Met1‐/Lys63‐linked ubiquitination induces NF‐κB essential modulator (NEMO) compartmentalization to effectively activate NF‐κB signaling.^[^ ^178^ ^]^ MAVS activation and aggregation are promoted by K63‐linked ubiquitination upon viral infection.^[^ ^179^ ^]^ Therefore, DUBs are rationally involved in the regulation of PRRs signaling as summarized in **Table** **3**.

**Table 3:** The roles of DUBs in immunity.

DUB	Effect	Target	Linkage	Rationale
TLRs signaling				
USP4	Inhibition	TRAF6	Lys63	Prevent NF‐κB and AP1 signaling^[^ ^187^ ^]^
USP10	Inhibition	TRAF6	Lys63	Terminate NF‐κB signaling^[^ ^530^ ^]^
	Inhibition	NEMO	Met1	Terminate NF‐κB signaling^[^ ^531^ ^]^
USP18	Inhibition	TAK1, NEMO	Lys63	Inhibit TLR/NF‐κB signaling^[^ ^182^ ^]^
USP19	Inhibition	TRIF	Lys27	Inhibit TLR3/4 signaling^[^ ^532^ ^]^
USP25	Activation	TRAF3/6	Lys48	Stabilize TRAF3, promote Antiviral Res..ponses^[^ ^190^, ^191^ ^]^
OTUB1/2	Inhibition	TRAF3/6	Lys63	Inhibit Antiviral Res..ponses^[^ ^533^ ^]^
OTUD1	Inhibition	TRAF3/6	Lys48	Stabilize Smurf1, and promotes the Smurf1‐mediated degradation of MAVS, TRAF3/6^[^ ^534^ ^]^
		RIPK1	Lys63	Inhibit RIPK1‐mediated NF‐κB signaling to suppress colonic inflammation^[^ ^185^ ^]^
OTUD4	Inhibition	MyD88	Lys63	Inhibit TLR‐mediated NF‐κB signaling^[^ ^186^ ^]^
OTUD7B	Inhibition	TRAF3	Lys48	Prevent aberrant non‐canonical NF‐κB activation^[^ ^189^ ^]^
MYSM1	Inhibition	TRAF3/6	Lys63	Terminate TLR‐induced Antiviral Res..ponses^[^ ^535^ ^]^
A20	Inhibition	TRAF6	Lys63	Terminate TLR/NF‐κB activation^[^ ^180^ ^]^
		NEMO	N/A	Block IKK phosphorylation, or inhibiting NF‐κB activation^[^ ^181^, ^536^ ^]^
UCHL1	Inhibition	TRAF3	Lys63	Inhibit p65 phosphorylation and NF‐κB signaling^[^ ^188^ ^]^
CYLD	Inhibition	NEMO	Met1	Inhibit NF‐κB signaling^[^ ^184^ ^]^
RLRs signaling				
USP3	Inhibition	RIG‐I	Lys63	Inhibit type I IFN signaling^[^ ^207^ ^]^
USP4	Activation	RIG‐I	Lys48	Stabilize RIG‐I, sustain IFN induction^[^ ^214^ ^]^
	Activation	TRAF6	Lys48	Stabilize TRAF6, inhibit EV71 replication^[^ ^537^ ^]^
USP5	Inhibition	RIG‐I	Lys11/48	Induce RIG‐I degradation^[^ ^233^ ^]^
USP14	Inhibition	RIG‐I	Lys63	Inhibit RIG‐I‐triggered type I IFN signaling^[^ ^208^ ^]^
USP15	Inhibition	RIG‐I	Lys63	Inhibit RIG‐I signaling^[^ ^209^ ^]^
	Activation	TRIM25	Lys48	Stablize TRIM25, then enhance the TRIM25‐ and RIG‐I‐dependent type I IFN production^[^ ^212^ ^]^
USP17	Activation	RIG‐1	Lys48	Stabilize RIG‐I, boost Antiviral Res..ponses^[^ ^213^ ^]^
USP21	Inhibition	RIG‐I	Lys63	Inhibit RIG‐I‐dependent Antiviral Res..ponses^[^ ^210^ ^]^
USP27X	Inhibition	RIG‐I	Lys63	Inhibit RIG‐I‐dependent Antiviral Res..ponses^[^ ^211^ ^]^
CYLD	Inhibition	RIG‐I, TBK1	Lys63	Inhibit RIG‐I‐dependent Antiviral Res..ponses^[^ ^206^ ^]^
OTUD2	Inhibition	MAVS	Lys63	Inhibit IRF3, p65 activation, and IFN‐β production^[^ ^538^ ^]^
OTUD3	Inhibition	MAVS	Lys63	Inhibit Antiviral Res..ponses^[^ ^539^ ^]^
OTUD4	Activation	MAVS	Lys48	Stablize MAVS, induce antiviral signaling^[^ ^215^ ^]^
cGAS signaling				
USP1	Activation	TBK1	Lys48	Stablize TBK1, enhance IFN‐β secretion^[^ ^540^ ^]^
USP2	Inhibition	TBK1	Lys63	Inhibit TBK1 activation^[^ ^541^ ^]^
USP7	Inhibition	TBK1	Lys48	Stabilize TRIM27, inhibit type I IFN signaling^[^ ^542^ ^]^
	Activation	p65	Lys48	Stabilize p65^[^ ^543^ ^]^
USP13	Inhibition	STING	Lys27/33	Disrupt the interaction between STING and TBK1, inhibit Antiviral Res..ponses^[^ ^231^ ^]^
USP14	Activation	cGAS	Lys48	Stabilize cGAS, facilitate antiviral signaling^[^ ^222^ ^]^
USP20	Activation	STING	Lys48	Stabilize STING, promote Antiviral Res..ponses^[^ ^225^ ^]^
USP21	Inhibition	STING	Lys27/63	Inhibit type I IFN production^[^ ^232^ ^]^
USP22	Inhibition	STING	Lys27	Cooperate with USP13, inhibit Antiviral Res..ponses^[^ ^233^ ^]^
	Activation	IRF3	Lys48	Stabilize importin KPNA2 to facilitate IRF3 nuclear translocation^[^ ^544^ ^]^
USP27X	Activation	cGAS	Lys48	Stabilize cGAS, govern DNA‐mediated signaling^[^ ^223^ ^]^
USP29	Activation	cGAS	Lys48	Stabilize cGAS, facilitate antiviral signaling^[^ ^545^ ^]^
USP38	Inhibition	TBK1	Lys33	Promote TBK1 degradation through NLRP4 signalosome^[^ ^546^ ^]^
USP44	Activation	STING	Lys48	Stabilize STING, facilitate antiviral signaling^[^ ^227^ ^]^
USP49	Inhibition	STING	Lys63	Inhibit STING aggregation and TBK1 recruitment^[^ ^230^ ^]^
A20	Inhibition	TBK1	Lys63	Cooperate with TAX1BP1 to disrupt TRAF3‐TBK1‐IKKi signaling complex^[^ ^547^ ^]^
OTUD1	Inhibition	IRF3	Lys6/63	Inhibit IRF3 nuclear translocation and DNA binding capacity^[^ ^548^, ^549^ ^]^
CYLD	Activation	STING	Lys48	Stabilize STING, facilitate antiviral signaling^[^ ^226^ ^]^
Th1 and Th17 responses				
Trabid	Activation	JMJD2D	Lys29	Promote the production of the Th1 and Th17 subsets of inflammatory T cells^[^ ^237^ ^]^
Cezanne	Activation	ZAP70	Lys48	Facilitate ZAP activation and inducing TCR signaling^[^ ^239^ ^]^
USP4	Activation	RORγt	Lys48	Stabilize RORγt, induce rheumatic heart disease^[^ ^236^ ^]^
USP10	Activation	T‐bet	Lys48	Stabilize T‐bet in Th1 cells, induce asthma^[^ ^240^ ^]^
USP17	Activation	RORγt	Lys48	Positive regulator of RORγt in Th17 cells^[^ ^550^, ^551^, ^552^ ^]^
USP18	Activation	TAK1	Lys63	Induce differentiation of Th17 cells and cytokines secretion^[^ ^238^ ^]^
Treg responses				
USP7	Inhibition	FOXP3	Lys48	Stabilize FOXP3 in Treg cells, inhibit TNFα‐stimulated NF‐κB activity^[^ ^256^, ^543^, ^553^ ^]^
USP21	Activation	GATA3	Lys48	Stabilize GATA3 in Treg cells, limit inflammatory responses^[^ ^543^, ^553^ ^]^

### 4.1 DUBs in TLRs Signaling

TLRs are the most extensively researched PRRs (Figure 8A). They are inhibited by several DUBs through deubiquitylating NF‐κB signaling factors such as NEMO, receptor‐interacting serine/threonine‐protein kinase 1 (RIPK1), and tumor necrosis factor (TNF) receptor‐associated factor 6 (TRAF6).^[^ ^150^ ^]^ A20 is the best‐identified DUB associated with inflammation. A20 expression is promoted by the NF‐κB pathway and cytokine receptors. In turn, A20 can be part of a negative feedback mechanism inhibiting continued NF‐κB activation, as described above.^[^ ^150^, ^152^ ^]^ Intriguingly, the role of A20 is not straightforward since it contains an OTU domain and seven ZnF motifs with DUB activity and E3 Ub ligase activity. A20 removes Lys63‐linked polyubiquitin chains from TRAF6, which leads to the termination of TLR‐mediated signaling activity.^[^ ^150^, ^180^ ^]^ In addition, A20 interacts with ubiquitinated NEMO by its ZnF motifs, which blocks IKK phosphorylation triggered by upstream TGF‐β‐activated kinase 1 (TAK1), and inhibits NF‐κB activation.^[^ ^181^ ^]^ USP18 exerts a negative regulatory effect on NF‐κB signaling by employing distinct mechanisms to target TAK1 and NEMO for deubiquitination.^[^ ^182^ ^]^ CYLD also negatively modulates TLRs signaling by removing Lys63‐ and Met1‐linked Ub‐chain from RIPK1, TRAF2, and NEMO.^[^ ^61^, ^183^, ^184^ ^]^ OTUD1 physically interacts with RIPK1 and specifically cleaves Lys63‐linked ub‐chain from RIPK1, thereby inhibiting the recruitment of NEMO.^[^ ^185^ ^]^ OTUD4 exerts Lys63‐specific DUB activity for myeloid differentiation primary response 88 (MyD88), which is indispensable for all TLRs involved in antiviral immunity, except TLR3.^[^ ^126^ ^]^ OTUD4 negatively regulates the TLR‐induced NF‐κB pathway by targeting MyD88. The affinity of OTUD4 to Lys63‐linked chains on MyD88 is increased by an adjacent UIM.^[^ ^186^ ^]^

USP4 is a negative regulator for TLR/IL‐1R signaling and subsequent immune responses by deubiquitinating TRAF6, which is essential for governing downstream TLR signaling.^[^ ^187^ ^]^ Additionally, USP4 depletion in growing zebrafish larvae induces pro‐inflammatory cytokines and increases susceptibility to lipopolysaccharide (LPS) challenge.^[^ ^187^ ^]^ UCHL1 removes Lys63‐linked Ub‐chain on TRAF3 (another essential regulator in TLR signaling), which decreases the production of pro‐inflammatory cytokines, chemokines, and type I IFN in response to high‐risk human papillomavirus (hrHPV) infection.^[^ ^188^ ^]^ TRAF3 is also targeted by OTUD7B through the Lys48‐specific DUB function, which restrains TRAF3 proteolysis and blocks abnormal non‐canonical NF‐κB activity.^[^ ^189^ ^]^ Thus, TRAF3 can be deubiquitinated by multiple DUBs and performs distinct effects during viral infection. Intriguingly, USP25 induced by viral infection prevents TRAF3 and TRAF6 from degradation. USP25 knockout mice are more susceptible to viral infection compared with the normal mice.^[^ ^190^, ^191^ ^]^ USP25 is the only identified DUB that plays a positive regulatory role in the TLRs signaling till now (Table 3).

### 4.2 DUBs in RLRs Signaling

RLRs can effectively fill in the gaps of most nonimmune cells due to low TLR expression during antiviral immune responses (Figure 8B). RLRs are cytosolic RNA sensors, which include three members: RIG‐I,^[^ ^192^ ^]^ melanoma differentiation‐associated protein 5 (MDA5),^[^ ^193^ ^]^ and laboratory of genetics and physiology 2 (LGP2).^[^ ^194^ ^]^ Lys63‐linked Ub‐chain induced by E3 ligases tripartite motif 25 (TRIM25), RNF135, TRIM4, and TRIM31 promote the stability and oligomerization of RLRs, thereby promoting downstream signaling.^[^ ^179^, ^195^, ^196^, ^197^, ^198^, ^199^ ^]^ However, degradative Lys48‐ and Lys27‐linked Ub‐chain mediated by E3 ligases RNF125, RNF122, CHIP, STUB1, and TRIM40 promote the degradation of RIG‐I and MDA5 to negatively regulate the signaling cascades.^[^ ^200^, ^201^, ^202^, ^203^, ^204^ ^]^ CYLD is the first DUB confirmed to deubiquitinate RIG‐I through cleavage of its Lys63‐linked Ub‐chain which disrupts the fundamental activation and impairs IFNs production.^[^ ^205^, ^206^ ^]^ USP3 serves as a negative regulator of RIG‐I signaling via trimming Lys63‐linked Ub on RIG‐I. upon viral infection or ligand stimuli, USP3 interacts with the CARD domain of RIG‐I and cleaves Lys63‐linked Ub on RIG‐I.^[^ ^207^ ^]^ However, the detailed mechanism by which USP3 governs RIG‐I in vivo remains unclear. USP14,^[^ ^208^ ^]^ USP15,^[^ ^209^ ^]^ USP21,^[^ ^210^ ^]^ and USP27X,^[^ ^211^ ^]^ also negatively regulate RIG‐I signaling through removal of Lys63‐linked Ub on RIG‐I.

Whereas, USP15 exerts a positive regulation of RIG‐I signaling. E3 ligase TRIM25 is in charge of Lys63‐linked poly‐ubiquitination on RIG‐I. USP15 counteracts the LUBAC‐induced degradation of TRIM25, which then contributes to continuing type I IFNs expression.^[^ ^212^ ^]^ Therefore, USP15 executes Janus‐faced effects by recognizing diverse substrates or antagonizing the effects of E3 ligase, thereby revealing the rigorous regulation of cellular immune homeostasis. USP17 removes Lys48‐linked Ub‐chain on RIG‐I or MDA5 to facilitate the expression of type I IFNs and Antiviral Responses.^[^ ^213^ ^]^ USP4 is another DUB responsible for promoting RIG‐I stability which increases RIG‐I‐induced IFN‐β signaling and reduces VSV replication. Moreover, USP4 protects TRAF6 from degradation and reduces enterovirus 71 (EV71) replication.^[^ ^214^ ^]^ DUB screening and biochemical analyses showed that OTUD4 removes Lys48‐linked Ub‐chain of MAVS, thereby limiting MAVS proteasomal degradation and activating downstream signaling in response to viral infection.^[^ ^215^ ^]^

### 4.3 DUBs in cGAS‐STING Signaling

cGAS is the primary pathway governing the immune response to pathogenic DNA in the cytosol (Figure 8C). Recognition of pathogenic DNA by cGAS activates cGAS to catalyze cGAMP formation.^[^ ^216^, ^217^ ^]^ cGAMP binds to the endoplasmic reticulum (ER)‐localized adaptor stimulator of IFN genes (STING), which promotes STING dimerization and to perinuclear Golgi.^[^ ^218^, ^219^ ^]^ TANK binding kinase 1 (TBK1) or IκB kinase (IKK) is then activated by STING, which stimulates phosphorylation and nuclear translocation of the transcription factor IFN regulatory factor 3/7 (IRF3/7), and to a lesser extent nuclear factor‐κB (NF‐κB) for production of pro‐inflammatory cytokines.^[^ ^220^, ^221^ ^]^

Lys48‐linked ubiquitination plays a negative role in the cGAS‐STING pathway by promoting proteasomal degradation of cGAS and STING.^[^ ^222^ ^]^ USP14 is recruited by E3 ligase TRIM14 to remove the Lys48‐linked Ub‐chain of cGAS and increase its stability. TRIM14 knockout mice are vulnerable to herpes simplex virus‐1 (HSV‐1) infection through damaging type I IFN production.^[^ ^222^ ^]^ USP27X directly binds to cGAS and trims Lys48‐linked Ub‐chain during viral infection.^[^ ^211^, ^223^ ^]^ Cleavage of the Lys48‐linked Ub‐chain in STING can be executed by diverse DUBs. USP20 directly interacts with‐ and cleaves Lys48‐linked Ub‐chain from STING after HSV‐1 infection, thus increasing the stability of STING and inducing cellular Antiviral Res..ponses.^[^ ^224^ ^]^ The catalytic activity of USP20 is enhanced by the recruitment of USP18.^[^ ^225^ ^]^ Other DUBs, such as CYLD, USP44, are also have positive regulation on STING during DNA virus infection.^[^ ^206^, ^226^, ^227^, ^228^ ^]^ USP44‐induced regulation of STING signaling is independent of USP20 and CYLD, suggesting that there is no functional redundancy between the three DUBs.^[^ ^227^ ^]^

Lys27‐, Lys33‐, and Lys63‐linked ubiquitination are implicated in the positive regulation of cGAS‐STING pathway by enhancing dimerization and enzymatic activity of cGAS and STING in response to pathogen DNA.^[^ ^229^ ^]^ USP49 removes Lys63‐linked Ub‐chain on STING following HSV‐1 stimulation, which contributes to decreased interactions between STING and TBK1 and inhibition of Antiviral Responses.^[^ ^230^ ^]^ In addition, USP13 deconjugates Lys27‐/Lys33‐linked Ub‐chain on STING which also disrupts the interaction between STING and TBK1.^[^ ^231^ ^]^ USP13 knockout mice are not susceptible to HSV‐1 infection.^[^ ^231^ ^]^ USP21 is recruited to STING by p38‐mediated phosphorylation of USP21 at the late stage of HSV‐1 infection. Then, USP21 negatively regulates the DNA virus‐induced production of type I IFNs by cleaving Lys27‐/Lys63‐linked Ub‐chain on STING.^[^ ^232^ ^]^ Furthermore, the Lys27‐linked Ub‐chain on STING is removed by the collaboration of USP22 and USP13 to regulate STING signaling negatively.^[^ ^233^ ^]^ Taken together, cGAS‐STING signaling can be elaborately regulated by multiple DUBs to enhance or impair innate Antiviral Res..ponses more accurately (Table 3).

### 4.4 DUBs in Inflammatory and Autoimmune Disorders

Chronic inflammatory and autoimmune diseases are generally characterized by a prolonged and persistent pro‐inflammatory state, such as metabolic syndrome, neurodegenerative disease, cardiovascular disease, rheumatoid arthritis (RA), and systemic lupus erythematosus (SLE).^[^ ^234^ ^]^ PRR stimulation causes dendritic cells (DCs) to secrete miscellaneous cytokines that regulate the differentiation of CD4^+^ T cells to different subclasses of T helper (Th) cells, including inducible regulatory T cells (Tregs), T follicular helper cells (Tfh), and Th1, Th2, Th9, and Th17 cells. Th17 cells execute pro‐inflammatory functions via the secretion of pro‐inflammatory cytokines including Interluekin‐17A (IL17A), IL‐17F, and IL‐22.^[^ ^235^ ^]^

USP4 promotes the stability of nuclear receptor retinoid‐elated orphan receptor‐γt (RORγt) in activated Th17 cells, then induces the production of IL‐17A.^[^ ^236^ ^]^ Expression of USP4, IL‐17A, and IL‐17F significantly increases in CD4^+^ T cells from RA patients compared with healthy volunteers.^[^ ^236^ ^]^ TRAF‐binding domain (Trabid) is essential for TLR‐regulated expression of IL‐12 and IL‐23 in DCs.^[^ ^237^ ^]^ Trabid deubiquitylates and stabilizes lysine demethylase 4D (KDM4D), which promotes histone modification by recruiting the NF‑κB family member REL to the *Il12* and *Il23* promoters.^[^ ^237^ ^]^ Depletion of Trabid in DCs inhibits the production of IL‐12 and IL‐23 and the generation of Th1 and Th17 cells, which neutralizes the stimulation of experimental autoimmune encephalomyelitis (EAE) in mice.^[^ ^237^ ^]^ USP18 is also connected with the activity of Th17 cells by promoting the TAK1‐TAK1‐associated binding protein (TAB) interaction; which is fundamental for Th17 cell differentiation and the autoimmune response.^[^ ^238^ ^]^

Moreover, T cell receptor signaling can be governed by Cezanne which deubiquitinates ZAP10 and disrupts the interaction between ZAP10 and negative regulatory phosphatases.^[^ ^239^ ^]^ ZAP70 phosphorylation is indispensable for complete activation and downstream phosphorylation of substrates, which enhances T cell signaling. youthful mice with *Cezanne* knockout have similar naïve‐ and memory‐like T cells compared with normal mice. However, IFN‐γ production in Th1 cells of elderly *Cezanne*‐deficient mice is reduced.^[^ ^239^ ^]^ This demonstrates that the production of pro‐inflammatory cytokines regulated by Cezanne in Th1 cells may be influenced by age stage to varying degrees, but the detailed mechanism remains unclear. T‐bet is critical in the development, differentiation, and function of Th1 cells. USP10 directly binds and inhibits T‐bet ubiquitination. *USP10* and *IFNG* mRNA expression is upregulated in asthmatic patient PBMC, suggesting USP10 may maintain a high level of T‐bet and IFN‐γ to fight against Th2‐dominated inflammation.^[^ ^240^ ^]^

Consequently, various DUBs exert multiple effects on distinct immune cells to cope with complex inflammation and immune responses. This suggests that building genetic models and developing inhibitors for these DUBs is conducive to discovering their therapeutic potential.

## 5 Virus‐Encoded DUBs in Innate Immunity

Identification and characterization of DUBs encoded by diverse virus families have provided valuable insights into how the viruses deal with elaborate host antiviral mechanisms through perturbing cellular ubiquitin‐dependent processes. Mainly, various signaling molecules of innate immune pathways can be targeted by virus‐encoded DUBs to suppress antiviral immunity and support viral replication.

Most viral DUB activity is mediated by papain‐like proteases (PLP or PLpro). The first identified viral DUB is adenovirus cysteine protease (Avp), which possesses the ability to cleave Lys48‐linked tetraUb chains and ISG15.^[^ ^241^, ^242^ ^]^ Structural analysis revealed that Avp active site comprises residues His54, Glu71, and Cys122, which closely align with the catalytic triad of papain.^[^ ^241^ ^]^ Nairovirus Crimean‐Congo hemorrhagic fever (CCHFV) is a tick‐borne virus and is widely distributed. Individuals infected with CCHF are at risk of multi‐organ failure and hemorrhaging.^[^ ^243^ ^]^ The OTU DUB of CCHFV has been identified having deubiquitinating enzyme activity, specifically targeting Lys48 and Lys63 Ub‐chain, as well as ISG15 from cellular proteins.^[^ ^244^ ^]^ The structure study revealed that the active site of CCHFV OUT consisted of a catalytic triad, comprising Cys‐His‐Asp residues, arranged in a geometrically competent conformation for catalysis.^[^ ^245^ ^]^ The CCHFV OTU was found to suppress IFN‐β response by removing ubiquitin (Ub) from RIG‐I, IRF3.^[^ ^246^ ^]^

Furthermore, the PLpro of severe acute respiratory syndrome coronavirus (SARS‐CoV) possesses deubiquitinating enzyme function and deISGylation activities.^[^ ^247^ ^]^ A crystal structure analysis of SARS‐CoV PLpro has confirmed its domain organization to be similar to that of human USP7, comprising “thumb,” “palm,” and “fingers” subdomains that assemble together in a configuration resembling an extended right hand.^[^ ^248^ ^]^ The PLpro enzyme exhibits activity towards IRF3 Lys48‐linked Ub‐chain and is implicated in the suppression of cellular innate immune responses.^[^ ^249^, ^250^ ^]^ In addition, SARS‐CoV PLpro can also removing Lys63‐linked Ub‐chain of TRAF3 and TRAF6, thereby suppresses the TLR7 signaling and the production of IFNs and pro‐inflammatory cytokines.^[^ ^251^ ^]^ Seneca Valley Virus (SVV) 3C protease inhibiting the IFN‐β production and promotes virus replication by eliminating ubiquitination of RIG‐I, TBK1, and TRAF3.^[^ ^252^ ^]^

Some virus‐encoded DUBs can negatively act on STING and NF‐κB signaling to manipulate anti‐viral immune responses. Herpes simplex virus (HSV) UL36USP can block type I IFN production by directly interacting with and deubiquitinating Lys48‐linked Ub‐chain of STING.^[^ ^253^ ^]^ Human cytomegalovirus (HCMV) pUL48 inhibits PRR‐induced type I IFN signaling through deubiquitinating several key molecules, such as STING, TRAF3, TRAF6, IRAK1, and IRF7.^[^ ^254^ ^]^ Epstein‐Barr virus (EBV) large tegument protein, BPLF1, deubiquitinates IκBα, TRAF6, and NEMO, thereby suppressing the NF‐κB signaling activation at multiple levels.^[^ ^255^ ^]^

A growing number of virus‐encoded DUBs have been identified and are recognized to play a pivotal role in antagonizing host immune system as listed in **Table** **4**. Virus‐encoded DUB‐mediated interaction between virus and host provides a deeper insight into viral pathogenic mechanism. Drugs targeting virus‐encoded DUB has drawn attention towards counteracting virus infection. In addition, harnessing viral DUBs for oncolytic use could be a particular interesting direction for future exploration.

**Table 4:** Virus‐encoded DUBs in host innate antiviral immunity.

Virus family	Virus	Virus‐encoded DUB	Host Target	Linkage	Rationale
RNA virus					
Coronaviridae/ alphacoronaviruses	Human coronavirus NL63	PLP2	RIG‐I, TBK1, IRF3, STING	Lys48/63	Negatively regulate antiviral defenses by disrupting the STING‐mediated IFN induction^[^ ^554^ ^]^
	Mouse hepatitis virus	MHV PLP2	IRF3, TBK1	NA	Negatively regulate type I IFN signaling^[^ ^555^, ^556^ ^]^
	Porcine epidemic diarrhea virus	PEDV PLP2	RIG‐I, STING	NA	Interfere with the RIG‐I‐ and STING‐mediated signaling^[^ ^557^ ^]^
Coronaviridae/ betacoronaviruses	SARS‐coronavirus (2)	SARS‐CoV PLpro	RIG‐I, TRAF3, STING, TBK1, IRF3, MDA5	Lys48/63, ISG15	Negatively regulate IRF3 activation.^[^ ^558^, ^559^ ^]^ Antagonize ISG15‐dependent activation of MDA5^[^ ^560^ ^]^
	MERS‐ coronavirus	MERS‐CoV PLpro	IRF3	Lys48/63, ISG15	Negatively regulate IRF3 activation^[^ ^561^, ^562^ ^]^
Arteriviridae	Porcine reproductive and respiratory syndrome virus	PRRSV PLP2	RIG‐I	Lys6/11/27/29/33/48/63	Inhibits RIG‐I‐mediated IFN signaling^[^ ^246^ ^]^
			IκBα		Inhibit NF‐κB activation^[^ ^563^ ^]^
	Equine arteritis virus	EAV PLP2	RIG‐I	NA	Inhibits RIG‐I‐mediated IFN signaling^[^ ^246^ ^]^
Bunyaviridae/ nairoviruses	Crimean‐Congo hemorrhagic fever virus	CCHFV OTU	MDA5, RIG‐I	Lys6/11/48/63, ISG15	Inactivate RLR‐mediated innate immune signaling^[^ ^246^ ^]^
Arteriviridae	Simian hemorrhagic fever virus	SHFV PLP2	RIG‐I	NA	Inhibits RIG‐I‐mediated IFN signaling^[^ ^246^ ^]^
	Lactate dehydrogenase‐elevating virus	LDV PLP2	RIG‐I	NA	Inhibits RIG‐I‐mediated IFN signaling^[^ ^246^ ^]^
Picornaviridae	Dugbe virus	DUGV OTU	NA	Lys48/63	Negatively regulate type I IFN signaling^[^ ^564^ ^]^
	Foot‐and‐mouth disease virus	FMDV Lbpro	RIG‐I, TBK1, TRAF3, TRAF6	Lys48/63	Negatively regulate type I IFN signaling^[^ ^565^ ^]^
	Seneca valley viru	SVV 3Cpro	RIG‐I, TBK1, TRAF3	NA	block IFN‐β induction^[^ ^252^ ^]^
DNA virus					
Adenoviridae	Adenovirus	Adenain	Histone H2A	Lys48, ISG15	Acquire an advantageous property by adenovirus^[^ ^241^ ^]^
Herpesviridae/ alphaherpesviruses	Herpes simplex virus	UL36USP	TRAF3	Lys48/63	Deubiquitinate TRAF3 and prevent the recruitment of the downstream adaptor TBK1^[^ ^566^ ^]^
			IκBα	Lys48	Restrict IκBα degradation and finally abrogate NF‐κB activation^[^ ^567^ ^]^
			STING	Lys48	Inhibits induction of type I IFN^[^ ^253^ ^]^
Herpesviridae/ betaherpesviruses	Human cytomegalovirus	pUL36USP	MLKL	Lys63	Degrade MLKL and inhibit necroptosis^[^ ^568^ ^]^
Herpesviridae/ gammaherpesviruses	Epstein‐Barr virus	BPLF1	PCNA	Lys48/63	Disrupt the cellular response to DNA damage^[^ ^569^ ^]^
			SQSTM1/p62		Regulate selective autophagy, which may promote infection and the production of infectious virus^[^ ^570^ ^]^
			Cullin		Benefit virus life cycle by inducing a replication‐permissive S‐phase‐like cellular environment^[^ ^571^ ^]^
			TRAF6		Deubiquitinate TRAF6 to inhibit NF‐κB signaling^[^ ^572^ ^]^
			TRAF6, NEMO, IκBα		Deubiquitinate signaling intermediates in the TLR cascade to counteract innate anti‐viral immunity^[^ ^255^ ^]^
	Kaposi's sarcoma‐associated herpesvirus	KSV ORF64	RIG‐I	Lys48/63	Suppresse RIG‐I‐mediated IFN signaling^[^ ^573^ ^]^
Herpesviridae	Hepatitis B virus	HBx	RIG‐I	Lys63	Evade the induction of IFN and IFN‐induced antiviral effects^[^ ^574^ ^]^
			RIG‐I, TBK1, CARDIF, TRIF, NEMO, IKKi, IRF3		Negatively regulate type I IFN production^[^ ^575^ ^]^

## 6 DUBs in the Cross‐Talk Between Cancer and Immune Response

The antitumor potential of therapeutically restraining DUB participation in the immune system is being diligently researched given the multiple roles of DUBs in the immune response. USP7 exerts a variety of effects in DNA repair, apoptosis, and tumor metastasis which largely depends on the distinct functions of protein substrates. USP7 stabilizes the expression of Forkhead box protein P3 (FOXP3), thus enhancing the regulatory T cell (Treg) suppressive capacity facilitated by this critical transcription factor.^[^ ^256^ ^]^ USP7 depletion prompts the polarization of TAMs (tumor‐associated macrophages) from M2 into M1 by stimulating the P38 MAPK pathway and enhancing the expression of immune checkpoint molecule programmed death ligand 1 (PD‐L1) in the tumor microenvironment.^[^ ^257^ ^]^ Accordingly, USP7 inhibition integrated with anti‐PD‐1 immunotherapy could exert a more effective inhibitory effect on tumors. In addition, USP7 and USP21 enhance GATA3‐regulated activity in FOXP3‐expressing cells.^[^ ^258^ ^]^ USP21 deficiency in Treg cells reduces FOXP3 expression, which reconciles the expression of Treg signature genes, and diminishes their suppressive activity.^[^ ^259^ ^]^ Treg cells limit antitumor immune responses and facilitate tumor survival, therefore, anticancer immunotherapies recommend FOXP3 depletion in Treg cells by targeting USP7 and USP21.

OTUB1 promotes immunosuppression of diverse cancers by targeting PD‐L1 and governs the transcription and post‐transcription of several tumor‐driving factors, including signal transducer and activator of transcription 3 (STAT3), NF‐κB, Hippo, phosphoinositide 3‐kinase (PI3K)/AKT, etc.^[^ ^260^, ^261^ ^]^ A cohort of cytokines and chemokines are induced by OTUB1, which recruit and modulate the immunosuppressive processes, resulting in cancer immune evasion.^[^ ^261^ ^]^ OTUB1 mediates the suppressive function of Treg cells, and the differentiation and maturation of B cells, T cells, and NK cells, which supports the potential role of OTUB1 in the tumor microenvironment.^[^ ^262^ ^]^ However, the specific function of OTUB1 and its regulatory network in different tumor microenvironments requires further exploration. Additionally, COP9 signalosome 5 (CSN5), USP8, USP21, and USP22 have been reported to deubiquitinate and stabilized PD‐L1 in cancer cells, thereby suppressing antitumor immunity.^[^ ^263^, ^264^, ^265^, ^266^ ^]^

DUBs‐regulated cross‐talk between tumorigenesis and immune response mainly focuses on the immunosuppression mediated by Treg cells and immune checkpoint. DUBs generally stabilize transcriptional factors to promote the expression of Treg signature genes and tumor‐driving factors. However, the antitumor potential of DUBs in B cells, T cells, and NK cells is relatively limited. Remarkably, as mentioned above, DUBs play a broad regulatory role in various immune signaling cascades, which reveals that there is great potential for exploration in the crosstalk governed by DUBs between cancer and immunity.

## 7 Therapeutic Applications via Targeting DUBs

Mounting evidence of DUBs in the pathogenesis cancer and immune disorders has brought about the remarkable attractiveness for DUBs as potential therapeutic targets (**Table** **5**). There has been a growing rate of progress on successfully screening and identifying potent and specific small molecule inhibitors of DUBs in past years. This highlights that DUBs are certainly druggable and have potential therapeutic targets in diseases ranging from oncology to immunology. The strategy of DUB inhibition can be roughly grouped into two types: conformational stabilization in enzymatic domain and allosteric inhibition in non‐catalytic sites.

**Table 5:** Nonexhaustive DUBs inhibitors in anti‐cancer and inflammation.

DUB	Inhibitor	Characteristics of inhibition	Disease indication
USP1	ML323	Highly selective, reversible	Oncology^[^ ^274^ ^]^
	SJB2‐043	Highly selective	Oncology^[^ ^576^ ^]^
	SJB3‐019A	Highly selective, irreversible	Oncology^[^ ^577^ ^]^
	GW7647	Non‐selective, reversible	Oncology^[^ ^275^ ^]^
	Pimozide	Non‐selective, reversible	Oncology^[^ ^275^ ^]^
	Rottlerin	Non‐selective, irreversible	Oncology^[^ ^275^ ^]^
USP2	ML364	Non‐selective, reversible	Oncology^[^ ^578^ ^]^
	LCAHA 6TG	Non‐selective Non‐selective, irreversible	Oncology^[^ ^579^ ^]^ Oncology^[^ ^580^ ^]^
USP4	Vialinin A	Non‐selective	Inflammation and oncology^[^ ^236^, ^581^ ^]^
USP5	WP1130 Vialinin A	Non‐selective Non‐selective	Oncology^[^ ^296^, ^582^ ^]^ Inflammation and oncology^[^ ^236^, ^581^ ^]^
USP7	FT671 FT827 GNE‐6640 GNE‐6776 Compound 1 Compound 4 P5091 P22077 HBX41108 HBX19818 Cpd1 Cpd2 Cpd8 XL188 Ursolic acid	Highly selective Highly selective Highly selective Highly selective Non‐selective Highly selective Highly selective Non‐selective Non‐selective, irreversible Non‐selective, irreversible Highly selective Highly selective Highly selective Highly selective Non‐selective	Oncology^[^ ^269^ ^]^ Oncology^[^ ^269^ ^]^ Oncology^[^ ^583^ ^]^ Oncology^[^ ^583^ ^]^ Oncology^[^ ^584^ ^]^ Oncology^[^ ^585^ ^]^ Oncology, immuo‐oncology^[^ ^584^ ^]^ Oncology, immuo‐oncology^[^ ^586^, ^587^ ^]^ Oncology^[^ ^588^ ^]^ Oncology^[^ ^589^ ^]^ Oncology^[^ ^588^ ^]^ Oncology^[^ ^588^ ^]^ Oncology^[^ ^590^ ^]^
USP8	DUBs‐IN‐2 MB7295	Highly selective Highly selective	Oncology^[^ ^591^ ^]^ Oncology^[^ ^592^ ^]^
USP9X	WP1130 EOAI3402143	Non‐selective, reversible Non‐selective, reversible	Oncology^[^ ^582^ ^]^ Oncology^[^ ^404^ ^]^
USP10	Spautin 1	Non‐selective	Inflammation^[^ ^593^ ^]^
USP11	MIX	Non‐selective	Oncology^[^ ^268^ ^]^
USP13	Spautin 1	Non‐selective	Inflammation^[^ ^593^ ^]^
USP14	IU‐1/analogues b‐AP15 VLX1570 WP1130 Auranofin	Non‐selective Non‐selective, reversible Non‐selective, reversible Non‐selective Non‐selective	Oncology^[^ ^594^, ^595^, ^596^ ^]^ Oncology^[^ ^597^, ^598^ ^]^ Oncology^[^ ^599^ ^]^ Oncology^[^ ^582^ ^]^ Oncology^[^ ^600^ ^]^
USP15	MIX	Non‐selective	Oncology^[^ ^268^ ^]^
USP17	WP1130	Non‐selective	Oncology^[^ ^582^ ^]^
USP20	GSK2643943A	Highly selective	Oncology^[^ ^601^ ^]^
USP24	EOAI3402143	Non‐selective, reversible	Oncology^[^ ^404^ ^]^
USP25	AZ1/2/3/4	Non‐selective, reversible	Oncology^[^ ^602^ ^]^
USP28	AZ1/2/3/4	Non‐selective, reversible	Oncology^[^ ^602^ ^]^
USP30	FT385 MF094 MF095	Highly selective Highly selective Highly selective	Oncology^[^ ^603^ ^]^ Oncology^[^ ^603^ ^]^ Oncology^[^ ^603^ ^]^
USP47	Compound 1	Non‐selective	Oncology^[^ ^584^ ^]^
POH1	8‐thioquinoline Capzimin phen	Non‐selective Highly selective Highly selective	Oncology^[^ ^604^ ^]^ Oncology^[^ ^605^ ^]^ Oncology^[^ ^606^ ^]^
UCHL1	LDN‐57444	Highly selective	Oncology^[^ ^607^ ^]^
	GK13S	Highly selective	Oncology^[^ ^608^ ^]^
	6RK73	Highly selective, irreversible	Oncology^[^ ^169^ ^]^
UCHL5	IU‐1/analogues	Non‐selective	Oncology^[^ ^594^, ^595^, ^596^ ^]^
	b‐AP15 VLX1570 WP1130 Auranofin	Non‐selective, reversible Non‐selective, reversible Non‐selective Non‐selective	Oncology^[^ ^597^, ^598^ ^]^ Oncology^[^ ^599^ ^]^ Oncology^[^ ^582^ ^]^ Oncology^[^ ^600^ ^]^
Trabid	NSC112200	Highly selective	Inflammation and oncology^[^ ^609^, ^610^ ^]^
CYLD	Subquinocin	Non‐selective	Inflammation^[^ ^611^ ^]^
SARS‐CoV PLpro	GRL0617	Highly selective, reversible	Anti‐infection^[^ ^276^ ^]^
	DDL‐701	Highly selective, reversible	Anti‐infection^[^ ^279^ ^]^

DUB inhibitors maintain the target DUB in an inactive or active conformation by directly interacting with catalytic‐site domains. Antineoplastic agent mitoxantrone (MIX) interacts with USP15 catalytic histidine H862 in a non‐covalent manner, which keeps USP15 catalytic domain in an inactive conformation state.^[^ ^267^, ^268^ ^]^ Small molecule FT827 forms a covalent bond between its vinyl sulfonamide residue and the catalytic cysteine of USP7, thereby stabilizing USP7 in a catalytically inactivated conformation and disrupting the coupling between Ub and USP7.^[^ ^269^ ^]^ CSN5 contains a JAMM domain with a Zn^2+^ ion that is essential for its catalytic activity. The inhibitor CSN5i‐3 developed by Novartis has a zinc‐binding group for attachment onto the catalytic site, therefore stabilizing CSN5 in an active conformation.^[^ ^270^ ^]^ Moreover, VLX1570 is a ring‐expanded version of b‐AP15 that specifically impairs the activity of USP14 and UCHL5 in the 19S regulatory subunit; it is associated with unique conformations of these DUBs.^[^ ^271^ ^]^ b‐AP15 exhibits apoptosis‐inducing and antineoplastic activities in various solid tumor models, including multiple myeloma.^[^ ^272^, ^273^ ^]^

On the basis of extensive oncogenic functions of USPs family in various cancers, compounds selectively targeting the USPs have been developed to antagonizes their tumorigenic effects. ML323 exerts a selective and reversible effect on the catalytic activity of USP1 in an allosteric manner that misaligns the conformation of Ub‐binding motif.^[^ ^274^ ^]^ Unexpectedly, ML323 enhances cisplatin susceptibility in osteosarcoma and NSCLC cells through affecting the DNA damage response.^[^ ^274^ ^]^ Moreover, GW7647 and pimozide also restrain USP1 catalytic activity in a non‐selective way. Both of them are allosteric inhibitors outside of enzymatic activity sites of USP1.^[^ ^275^ ^]^ In addition to USP1, the catalytic activity of USP7 can also be suppressed by GW7647 and pimozide,^[^ ^275^ ^]^ but the detailed functional effects are unclear.

Allosteric inhibitors competitively interact with the Ub‐binding sites of DUBs and hinder substrate recognition. The small molecule GRL0617 was first reported as a competitive non‐covalent inhibitor for a papain‐like protease (PLpro) encoded by SARS‐CoV and MERS‐CoV.^[^ ^276^ ^]^ PLpro is a viral DUB that plays a vital role in the regulation of host cellular processes. GRL0617 blocks Ub access to the PLpro catalytic site by occupying the binding pocket for the distal Ub's C‐terminal residues. Additionally, several other inhibitors, such as rac3j, rac3k and rac5c targeting SARS‐CoV PLpro in clinical and preclinical stages have been described elsewhere.^[^ ^277^ ^]^ Among them, rac5c also have potent inhibitory effect on SARS2 PLpro, which might be deliberated as a potential therapeutic strategy for COVID‐19.^[^ ^278^ ^]^ Another small molecule DDL‐701 is also an effective SARS2 PLpro inhibitor which displayed sustained inhibitory activity.^[^ ^279^ ^]^

Although certain progress has been made in the development of drug targets for DUBs, it is still challenging for researchers to overcome a variety of existing obstacles. First, multiple DUBs are tightly related in similar catalytic pockets, which makes it difficult to develop potent inhibitors that represent selectivity for individual DUBs. Secondly, the interaction between DUB and substrate is not simply one‐to‐one. The same substrate may be targeted by various DUBs, while several DUBs may target multiple substrates. Noteworthily, DUBs exert diametrically opposed roles according to the complex context. Therefore, anti‐cancer efficacies and cytotoxicity for the development of DUB inhibitors should be attentively considered in future clinical trials. Finally, the mechanisms of DUBs regulation are generally complicated, including switch between active and non‐active conformations, allosteric effects, and substrate‐regulated catalysis. This makes it more difficult to carry out the primary screening and biochemical assays.

## 8 Concluding Remarks

This review summarized the multiple regulatory roles of DUBs in tumorigenesis and immunity. DUBs counteract the biological effects of E3 Ub ligases by removing the Ub chains from downstream substrates. Deubiquitination mediated by DUBs is accomplished alone or in combination with a complex to promote their enzymatic activities. Most DUBs confer some level of specificity regarding their action on diverse substrates or Ub‐linkage types. Indeed, there are still many gaps in knowledge of DUB specificity and mechanisms which limits the development of therapeutic applications in this field.

Various linkage types, lengths, and PTMs contribute to the miscellaneous architecture of polyubiquitin chains. Atypical linkage types of polyubiquitin chains may exert extremely distinctive effects on diverse cellular processes.^[^ ^280^ ^]^ However, the topology of polyubiquitin chains and the mechanism of DUB recognition remains unclear. There is a lack of effective tools and methods to reveal the architecture of polyubiquitin chains, which is necessary to precisely identify the cleavage specificity of DUBs on polyubiquitin chains. Furthermore, ubiquitination and deubiquitination usually occur simultaneously (temporally and spatially) due to the close functional interactions and reciprocity between E3 ligases and DUBs. It is worth to elucidate how E3 ligases and DUBs selectively cooperate with key signaling mediators to govern tumor and immune responses. Finally, DUBs are either cysteine proteases or metalloproteases. Therefore, it deserves to investigate whether other cysteine proteases or metalloproteases serve as DUBs to fine‐tune the Ub system and maintain homeostasis. Innovative Ub probes targeting metalloproteases, serine proteases, or aspartate proteases will be helpful to discover novel DUB classes.^[^ ^35^, ^281^ ^]^

Taken together, this review provides a glimpse into the Janus‐faced regulatory roles of DUBs in protein turnover and numerous signaling pathways, which is critical for the development of cancer and immunity. The development of innovative approaches will ultimately provide new insights into the mechanics of the ubiquitin system and confer DUBs with broad therapeutic potential.

## Conflict of Interest

The authors declare no conflict of interest.

## Author Contributions

J.R., P.Y., and S.L. contributed equally to this work. J.R., P.Y., and S.L. conceived and drafted the manuscript. J.R., P.Y., and S.L. drew the figures. J.R., R.L., X.N., Y.C., and P.Y. discussed the concepts of the manuscript. P.Y., S.L., and Y.W. collected the information and summarized tables. Z.Z., F.Z., and L.Z. provided valuable discussion and revised the manuscript. The authors apologize to those researchers whose related works were not cited in this review.
